# Unbiased distance correlation with sample-size-aware confidence bounds for comparative omics network analysis

**DOI:** 10.3389/fbinf.2026.1788010

**Published:** 2026-06-11

**Authors:** Miroslava Cuperlovic-Culf, Anuradha Surendra, Irina Alecu, Abdullah Mahdi, Finn Archinuk, Hosna Jabbari

**Affiliations:** 1 Digital Technologies Research Centre, National Research Council of Canada, Ottawa, ON, Canada; 2 Department of Biochemistry, Microbiology, and Immunology, Ottawa Institute of Systems Biology, University of Ottawa, Ottawa, ON, Canada; 3 Neurolipidomics Laboratory, Ottawa Brain and Mind Research Institute, University of Ottawa, Ottawa, ON, Canada; 4 Department of Biomedical Engineering, University of Alberta, Edmonton, AB, Canada

**Keywords:** Bernstein related inequalities, bioinformatics network analysis, bioinformatics software, correlation analysis, Hoeffding inequality, metabolomics analysis, sample size error estimate, unbiased distance correlation

## Abstract

**Introduction:**

Data-driven determination is a powerful approach for unbiased investigation of the functional relationships in biomolecular networks. Such networks can be inferred from omics data, where correlation analysis is a commonly used method. However, the correlation values depend strongly on sample variability and size of the sample set in the general case, leading to unstable results and possibly highly erroneous conclusions.

**Methods:**

In this work, we show that similar to the Pearson and Spearman correlation approaches, distance correlation as a general non-linear polytonic correlation method also depends on the sample size. We show that both the *p*- and correlation values decrease with increasing sample sizes independent of the type of functional relationship between the features but relative to the correlation level. However, the dependence on sample size is greatly reduced in the unbiased distance correlation formulation. We validate an equation to compute the *p*-value and propose a threshold based on the false discovery rate (FDR) to identify significant correlations in unbiased distance correlation. Furthermore, we derive an extension of Hoeffding’s inequality for estimating the error range of correlation as a function of the sample size as an additional sample-size-dependent confidence measure.

**Results:**

We integrated bias-corrected distance correlation with sample-size-matched bootstrapping, chi-squared *p*-value calculation, FDR adjustment, and empirical Bernstein/Hoeffding-type confidence bounds to support comparative correlation-network analysis in groups with unequal sample sizes. We also provide an online software solution for this application with extensive graphical presentations of the results. The use of this approach is demonstrated in the analysis of major network changes in Alzheimer’s disease (AD) using previously published metabolomics data. Our analysis shows major changes in the metabolic networks, with higher connectivity in the AD group than the control group and examples of network changes for specific metabolites.

**Conclusion:**

We present a method for unbiased distance correlation network derivation with permutations and comparisons between the sample groups. All approaches presented here are available through an online application (https://www.insilicobiology.ca/shiny/sidco+/), and all related code is available at https://github.com/computationalmetabolomicsca/sidco_plus.

## 1 Introduction

Data-driven determination of biomolecular networks is a powerful approach for unbiased investigations of functional relationships. Determination of the pairwise associations between features as edges between biomolecules offers an avenue for investigating their roles in various pathways and processes. Metabolomics and lipidomics, which entail high-throughput analyses of hydrophilic and lipophilic metabolites, provide measurements of the molecular abundances that could in turn offer insights into their complex relations through, for example, direct and indirect metabolic processes or signaling pathways. Specifically, determining and describing the metabolome network in a biological system is an essential step in identifying the affected enzymes, pathways, or signaling routes (Kennedy et al., 2025). Networks of biomolecules are routinely inferred from omics data as associations, correlations, or regulatory graphs (Zitnik et al., 2024) constructed from the behaviors or correlations derived from omics measurements. In this type of analysis, the molecules are perceived as the network nodes while the omics data are used to determine the presence, weights, and directionality of the edges (Li et al., 2024). This approach enables the inference of an implicit network based on common behaviors between nodes across samples.

Correlation analyses are broadly defined as statistical methodologies for establishing pairwise associations across multiple, potentially temporarily evolving signals; they remain among the major approaches for data-driven interaction analyses owing to their versatility and ease of interpretation. The Pearson and Spearman correlation-based methods are some of the commonly used techniques (Amara et al., 2022). While providing critical information about the level of dependencies, Pearson correlation measures only the linear and monotonic correlations that rely strongly on the assumption of normal distribution (Rosato et al., 2018). The Spearman method is a rank-based equivalent to Pearson correlation and measures the non-linear as well as discrete value relationships, albeit limited only the monotonic relationships. Several additional methods for network inference have been developed based on correlation approaches, as recently explored by Archinuk et al. (2025). For example, context likelihood of relatedness (CLR) (Faith et al., 2007) determines the mutual information between pairs of nodes while removing indirect relationships. The weighted gene correlation network analysis that has been previously applied to transcriptomic (Langfelder and Horvath, 2008) and metabolomics (Grapov et al., 2015; Pei et al., 2017) datasets is built on Pearson correlation with automatic determination of the threshold value based on the assumption of a scale-free network topology and user-defined power parameter (Langfelder and Horvath, 2007). However, the scale-free network structure assumption is not generally applicable to metabolic networks (Broido and Clauset, 2019), which leads to information loss (Lee et al., 2008). Further, different power parameter choices will produce distinct results. Some novel hybrid approaches were used to calculate the inverse Jacobian matrix from data-derived covariances and knowledge-based networks (Zitnik et al., 2024), which also rely strongly on the covariance metric. These strengths and weaknesses lead us to distance correlation, which is a non-parametric approach proposed as a measure of multiple types of data relationships, including correlations between vectors of different lengths (Edelmann et al., 2021; Gábor and Maria, 2009; Szekely and Rizzo, 2013a; Szekely and Rizzo, 2013b). Distance correlation considers the sparsity of omics data in general and has the potential to identify non-linear and polytonic relationships in non-normal distributions. By capturing these dependencies between molecular pairs, distance correlation preserves information about increasingly complex relationships, thereby enabling the determination of all covariance as well as establishing true independence between the variables (Richards et al., 2014). Several researchers (Tang et al., 2019; Cuperlovic-Culf et al., 2021) have employed distance correlation in metabolomics data analysis. Our research group (Monti et al., 2023) also provided a user-friendly solution for signed distance correlation calculations, which was applied in use cases by Svinin et al. (2025).

Correlation networks can be explicitly constructed to compare given sample types or conditions (e.g., healthy vs. diseased) and thus present a unique perspective on the molecular network of divergent states. However, regardless of the methodology, the correlation values are strongly influenced by the representation of population variance in each dataset and the lengths of the feature vectors thereof, i.e., sample sizes. Generally, larger samples lead to lower correlation levels. Population representation improves significantly with increase in sample size; this increase results in lower *p*-values, i.e., more statistically significant edges and denser networks, in larger datasets if the correlation threshold does not consider the sample size effects. Arguably, this is a known issue in all correlation approaches (Gomez-de-Mariscal et al., 2021; Kaplan et al., 2014). However, the bias introduced by limited sample sizes can potentially result in invalid correlation matrices, which is sometimes overlooked and results in erroneous conclusions (Lin, 2018).

The sample size necessary for convergence of the correlation values regardless of the correlation metric is still under evaluation. Using simulated data, Schönbrodt and Perugini (2013) demonstrated that the correlation values stabilize with approximately 250 samples in the set regardless of the magnitudes. However, Kretzschmar and Gignac (2019) determined that achieving stable and measurement-error-free correlations required approximately 490 samples. May and Looney (2022) proposed an approach to determine the optimal sample sizes for correlation level comparisons between conditions and reported that over 200 samples were required for each group. Together, these data emphasize that the exact number of samples required for stability depends on the size of the correlation, confidence level, and type of feature relationship used to achieve meaningful comparisons.

Beyond correlation stability, transitioning from the correlation matrix to a meaningful interaction network involves edge selection as the major consideration. Different threshold-based and tree-based approaches have been proposed as solutions for this purpose (Masuda et al., 2023); these methods range from the simplest approach, where values outside a user-defined threshold for either correlation or *p*-value are set to zero while values above the threshold are retained as-is, or the dichotomization approach wherein the values are set to one. In the proportional thresholding method, a fixed proportion of the strongest connections are retained for each group to ensure comparable edge density across sample types (Garrison et al., 2015). In the maximal spanning tree approach, the network correlation node pairs are added as edges sequentially to ensure that all nodes within a tree with *N* nodes and *N*-1 edges are connected. The sparsity of the tree formed thus is extreme; therefore, alternative approaches such as the planar maximally filtered graph have been proposed to create a network with 3*N*-6 edges, i.e., all possible edges (Tumminello et al., 2005). From the above works, the correlation value, significance level, and order of correlations are crucial in all methods. Thus, all approaches require assessment of the sample-size dependencies of the correlation values.

In the present work, we focus on sample-size-optimized threshold levels for the *p*-values and/or correlation levels to enable determination of the presence or absence of correlations appropriate for the number of samples available. In data sampling, the *p*-values approach zero in large sample sets regardless of the actual differences in data (Gomez-de-Mariscal et al., 2021). Gomez-de-Mariscal et al. (2021) showed the exponential dependence of *p*-value on the number of samples to argue justification of a minimal sample size and optimal threshold values. Herein, we expand this approach by investigating whether similar trends can be observed in distance correlation. We propose a method for unbiased distance correlation “power analysis” and establish a minimum sample size for a given correlation level along with a sample-size-appropriate threshold approach by combining *p*-value and confidence interval (CI) analysis; we also provide information about the minimum calculation error for a given sample size and probability. Furthermore, we derive an approximate method to determine the error range of distance correlation using approaches derived from the Hoeffding inequality analysis, specifically empirical Bernstein radii. It is important to highlight that in real-world omics experiments, notably with metabolomics measurements, the sample sizes are generally significantly below the theoretically required stability limits reported in empirical literature (Schönbrodt and Perugini, 2013; Kretzschmar and Gignac, 2019; May and Looney, 2022) and that comparative analyses are often performed between sample sets of different sizes. Our goal is to provide some considerations, recommendations, and methods to enable appropriate utilization of correlations in omics and particularly metabolomics research. These approaches are presented using a simulated dataset as well as previously published Alzheimer’s disease (AD) metabolomics measurements (Kalecký et al., 2022), where our method was used to investigate differences in correlation networks in the control and AD patient cohorts. All methods developed in this work are provided to the research community in an open-use and open-source application SIDCO+ at https://www.insilicobiology.ca/shiny/sidco+/.

## 2 Materials and methods

### 2.1 Distance correlation

*Original distance correlation* is calculated as a correlation of the distance covariances, as given in Equation 1:

$$R_{n}^{2}\left(X,Y\right)=\left\{\begin{array}{l} \frac{V_{n}^{2}\left(X,Y\right)}{\sqrt{V_{n}^{2}\left(X\right)V_{n}^{2}\left(Y\right)}}\;V_{n}^{2}\left(X\right)V_{n}^{2}\left(Y\right)>0,\\ 0\;{\;V}_{n}^{2}\left(X\right)V_{n}^{2}\left(Y\right)=0 \end{array}\right..$$ (1)

The distance covariance corresponding to the covariance in Pearson correlations is determined by Equation 2:

$$V_{n}^{2}\left(X,Y\right)=\frac{1}{n^{2}}\sum_{j=1}^{n}\sum_{k=1}^{n}A_{j,k}B_{j,k},$$ (2)

where *A* and *B* are doubly centered distance matrices calculated as simple linear functions of the pairwise distances between the elements in samples *X* and *Y*, respectively. Specifically, matrix *A* is given by Equation 3:

$$A_{j,k}=a_{j,k}-\frac{1}{n}\sum_{k=1}^{n}a_{j,k}-\frac{1}{n}\sum_{j=1}^{n}a_{j,k}+\frac{1}{n^{2}}\sum_{j,k=1}^{n}a_{j,k},$$

$$where\;a_{j,k}=\sqrt{\left(x_{j}-x_{k}\right){\left(x_{j}-x_{k}\right)}^{\prime }.}$$ (3)

Here, the distance between $x_{j}$ and $x_{k}$ is Euclidean. Similarly, matrix *B* is populated using equivalent measures for variable *Y*; in this case, we use the distance covariance defined above that follows V-statistics (Székely et al., 2007).

*Unbiased distance correlation* (used interchangeably with bias-corrected distance correlation) is used to correct the bias in the original distance correlation with the aim of having the estimator be equal to zero under full independence (Székely and Rizzo, 2013b) while following U-statistics (Huo and Székely, 2016) to provide faster calculations. In this process, the matrices *A* and *B* are recentered by subtracting the row and column means, adding the grand mean, and finally zeroing the diagonal. This step removes the marginal structures of *X* and *Y* while retaining only the joint structure. The main change in this case is that the doubly centered distance matrices become U-centered and are calculated as shown in Equation 4:

$${\tilde{A}}_{j,k}=\left\{\begin{array}{l} a_{j,k}-\frac{1}{n-2}\sum_{k=1}^{n}a_{j,k}-\frac{1}{n-2}\sum_{j=1}^{n}a_{j,k}+\frac{1}{\left(n-1\right)\left(n-2\right)}\sum_{j,k=1}^{n}a_{j,k},i\neq j\\ 0,i=j \end{array}\right..$$ (4)

Thus, double centering is achieved by subtracting the leave-one-out means instead of the direct means; thus, the expected distance covariance value is zero for fully orthogonal vectors. Furthermore, the unbiased distance correlation follows U-statistics with the distance covariance being equivalent to the inner product of the doubly centered distance matrices as $U_{n}^{2}\left(X,Y\right)=\left(\tilde{A}\cdot \tilde{B}\right)=\frac{1}{n-3}\sum_{k\neq l}{\tilde{A}}_{kl}{\tilde{B}}_{kl}$ , as proven previously (Huo and Székely, 2016; Monroy-Castillo et al., 2025). The variance formula is given by Equation 5:

$${\tilde{V}}_{n}^{2}\left(X,Y\right)=\frac{1}{n\left(n-3\right)}\sum_{k\neq j}^{n}{\tilde{A}}_{j,k}{\tilde{B}}_{j,k},$$ (5)

while the modified distance correlation is given by Equation 6:

$${\tilde{R}}_{n}\left(X,Y\right)=\left\{\begin{array}{l} \frac{{\tilde{V}}_{n}\left(X,Y\right)}{\sqrt{{\tilde{V}}_{n}\left(X\right){\tilde{V}}_{n}\left(Y\right)}}\;{\tilde{V}}_{n}\left(X\right){\tilde{V}}_{n}\left(Y\right)>0,\\ 0\;{\tilde{V}}_{n}\left(X\right){\tilde{V}}_{n}\left(Y\right)=0 \end{array}\right..$$ (6)

Importantly, unlike the original distance covariance, ${\tilde{V}}_{n}^{2}\left(X,Y\right)$ can have low negative values in extreme cases with very low covariance levels and small sample sizes. Thus, ${\tilde{R}}_{n}\left(X,Y\right)$ cannot be obtained in principle in these cases. Here, to provide computationally effective calculations for the edge case, we consider the truncating approach and set the negative values to zero rather than following a more complex approach (Monroy-Castillo et al., 2025) because our focus is not on determining true zero but providing a measure of the significant correlations.

### 2.2 CI and *p*-value calculations

For Pearson correlation, several authors such as Groppe (2017) have derived the CI for the normal distribution as a measure of the dispersion of the distribution around the mean value. Thus, the CI is defined as follows: $CI=z\pm \;\frac{z_{1-\frac{\alpha }{2}}+z_{1-\beta }}{\sqrt{n-3}}$ , where $z_{1-\frac{\alpha }{2}}\text{ and }z_{1-\beta }$ represent the standard normal distribution values corresponding to the risk of committing a type I error (*a*) and type II error (*b*), respectively. The Fisher z-transformation of correlation is defined as $z=\mathrm{atanh}\left(Cor\right)=0.5*\ln\left(\frac{1+Cor}{1-Cor}\right)$ and is used to stabilize the variance. The variance of the Fisher transformed correlation is given by $\frac{1}{\sqrt{n-3}}=\frac{z}{z_{1-\frac{\alpha }{2}}+z_{1-\beta }}$ when *CI =* 0. Following a straightforward reorganization of the formula, the number of samples *n* necessary to achieve a confident Pearson correlation level can be determined as shown in Equation 7 (Caycho-Rodríguez, 2020):

$$n={\left(2\frac{z_{1-\frac{\alpha }{2}}+z_{1-\beta }}{\ln\left(\frac{1\;+\;Cor}{1\;-\;Cor}\right)}\right)}^{2}+3.$$ (7)

As an example, for a desired CI of 95% and a statistical power of 80%, $z_{1-\frac{\alpha }{2}}=1.96\text{ and }z_{1-\beta }=0.84$ ; thus, a correlation value of 0.2 can be obtained with the desired confidence only if there are more than 194 samples available. For this case, Gnambs (2023) showed that the standard error estimation method reported by Bonett (2008) provides the least biased value. In Bonett’s model, the standard error is estimated as $1-{Cor}^{2}$ relative to the variance of the correlation distribution. The standard error estimate for a normally distributed correlation, such as the Pearson correlation, is further defined as $\sigma_{p}=\frac{1-{Cor}^{2}}{\sqrt{n-3}}$ (Bonett, 2008). Here, the *p*-value is calculated from the t statistic defined by $t=\frac{Cor\sqrt{n-2}}{\sqrt{1-{Cor}^{2}}}$ and number of degrees of freedom *df* = *n*–2.

For the V-statistic-based original distance correlation, the population-level target variance is $V^{2}\left(X,Y\right)=E\left[h\right]$ , with the kernel being defined as $h\left(\left(Y_{k}\right)\left(X_{l}Y_{l}\right)\right)=A_{k,l}B_{k,l}$ . The CI can be calculated using either bootstrap analysis or inequality formulas. We use the Bayesian bootstrap method (Rubin, 1981; Justus et al., 2024; Efron and Tibshirani, 1993) following the assumptions described in Justus et al. (2024). Here, for set of B samples, we simulate the parameters $\beta_{i}$ as a Dirichlet distribution of size *n* and define the mean and standard deviations of the values as follows: mean $\mu =\sum_{i=1}^{n}\beta_{i}{dcor}_{i}$ and standard deviation $\sigma =\sqrt{\sum_{i}^{n}{\beta_{i}\left({dcor}_{i}-\mu \right)}^{2}}$ . The CI is then determined from the generated bootstrap replicates as the 95% interval. The same analysis can provide the CIs for biased and unbiased distance correlations. The MATLAB (MathWorks Inc.) code for these is provided and used in the online version.

Alternatively, derivations based on Hoeffding’s inequality can be used to determine the distance covariance inequality for cases with no known distributions or any assumptions beyond boundedness. The general Hoeffding’s inequality for independent random variables is defined as in Equation 8a (Hoeffding, 1963):

$$\mathrm{Pr}\left(\frac{1}{n}\sum_{i=1}^{n}X_{i}-\frac{1}{N}\sum_{i=1}^{N}X_{i}\geq \varepsilon \right)\leq \exp\left(-\frac{2n\varepsilon^{2}}{{\left(b‐a\right)}^{2}}\right)\text{for }a=\min_{1\leq i\leq N}\;x_{i}\;and\;b=\max_{1\leq i\leq N}\;x_{i},$$ (8a)

where $X=(x_{1},\ldots \;x_{N}$ ) is the finite population of *N* real points and $(X_{1},\ldots \;X_{n}$ ) is a list of size *n* < *N* that is randomly sampled without replacement; $\frac{1}{N}\sum_{i=1}^{N}X_{i}$ is the expected population mean for $X_{i}$ , and $\frac{1}{n}\sum_{i=1}^{n}X_{i}$ is the mean value obtained for the *n* samples. For all ε>0 , the probability that the difference between the mean of the sampled values and expected value is smaller than ε is given by Equation 8a. This probability depends on the number of samples selected from the set, range of sample values, and error rate ε but does not depend on the distribution. For the properties resulting from sampling and property calculations without replacement using a kernel *g*(·) of multiplicity *h*, the corresponding Bernstein formula (Ai et al., 2023) is given by Equation 8b:

$$\mathrm{Pr}\left(U-\mu \geq \varepsilon \right)\leq \exp\left(-\frac{K\varepsilon^{2}}{2\left(\sigma^{2}+\frac{\left(b‐a\right)\varepsilon }{3}\right)}\right);\;\sigma^{2}=Var\left[g\left(X\right)\right];\;K=\lfloor n/h\rfloor .$$ (8b)

Here, we are looking for

$$\mathrm{Pr}\left(\left|{R_{n}}^{2}‐{R_{N}}^{2}\right|\leq \varepsilon \right)\geq 1‐\delta \;for\;all\;n,and\;R^{2}\in \left[0,1\right].$$

For the error measure, Maurer and Pontil (2009) used the empirical Bernstein radii, while Peel et al. (2010) derived an equivalent measure for the U-statistic case with probability of at least 1−δ and order 2m, as given by Equation 9:

ϵR2=2V^2X,Ylog⁡2/δnm+2⁡log⁡2/δ3n/m. (9)

For the correlation, the U-statistic order is 2 (with *m* = 1). V^2 can be calculated by bootstrapping; if this is not available, V^2=0 and only $\frac{2\log\left(2/\delta \right)}{3\left(n/m\right)}$ is used to compute the error. The non-asymptotic CI is then given by CIR2EB=max0,R^n2−ϵR2,min1,R^n2+ϵR2 along with δ . Finally, for unbiased distance correlation, the Fisher z-transform can be used for better finite-sample coverage, similar to the Pearson correlation example. For the unbiased distance correlation, the CI can be obtained by Equation 10:

$$CI=\tanh\left(Z\pm \frac{z_{1-\frac{\alpha }{2}}+z_{1-\beta }}{\sqrt{n-3}}\right),where\;Z=\mathrm{arctanh}\left(dCor_{unbiased}\right).$$ (10)

This CI calculation is general and does not involve any assumption for the dataset, thus providing very broad error bounds.

Distance correlation *p*-value: For biased distance correlation, the *p*-value is generally determined using the permutation test and calculated for B sampling operations as follows: *p-value = (Number of permutations where dCor* ^*2*^ *≥ dCor* ^*2*^_*n*_ *)/B.* Although this approach is applicable generally, it is inappropriate for very small sample sizes, and the result depends on the value of B; an increase in B generally leads to greater accuracy but at a major cost of computational time. Alternatively, for unbiased distance correlation, Shen et al. (2022) showed that the chi-squared test is a valid alternative to the permutation test; it was shown that the chi-squared test was a non-parametric and fast method appropriate for bias-corrected distance correlation and that it provided comparable results to the standard permutation test. Based on this approach, the *p*-value is given by Equation 11:

$$p=1-chi2cdf\left(nR_{n}^{*}\left(X,Y\right),1\right),$$ (11)

where chi2cdf is the chi-squared cumulative distribution function of degree 1 evaluated at $nR_{n}^{*}\left(X,Y\right)$ .

For unbiased distance correlation, Székely and Rizzo (2013a) showed a simple significance test following modified statistics for independence, which follows the Student’s t distribution while avoiding the need for permutation analysis. Székely and Rizzo (2013a) and Gao et al. (2021) also showed that the *p*-value for bias-corrected distance correlation for a large sample size of n→∞ and high-dimensional data can be calculated as $1-\Phi \left(T_{n}\right)$ , where $\Phi \left(\cdot \right)$ represents the standard normal distribution function and *T*_*n*_ is given by Equation 12:

$$T_{n}=\sqrt{\frac{n\left(n-3\right)}{2}-1\;}{\;R}_{n}^{*}\left(X,Y\right)/\sqrt{1-{R_{n}^{*}\left(X,Y\right)}^{2}}.$$ (12)

Here, $R_{n}^{*}\left(X,Y\right)$ is the bias-corrected distance correlation, and the X and Y values are mean-centered independent variables (Székely and Rizzo, 2013b). Gao et al. (2021) further showed that the accuracy of normal distribution approximation for distance correlation increases with increasing dimensionality of X and Y, showing that this approach can be used as a test of independence for correlation in this extreme case.

False discovery rate (FDR): This measure is used to control the expected proportion of false positive errors in the case of correlation analysis for a large feature set. In our application, we use the Benjamini and Hochberg (1995) method that maintains a significant change for the detection of true effects while controlling the proportion of false discoveries. The Benjamini–Hochberg method is used as the standard when independent of positively dependent values and is less conservative than the Benjamini and Yekutieli (2001) method; it is used here as a pragmatic screening correction. For a total of *M* hypothesis tests, *F* false discoveries (type I errors), and a total of *T* rejected null hypotheses, the FDR is defined as $FDR=Ε\left[\frac{F}{\max\left(T,1\right)}\right]$ . The Benjamini–Hochberg protocol implemented herein is as follows:

- Perform *M* hypothesis tests and rank the obtained *p*-values in ascending order as
$$p_{\left(1\right)}\leq p_{\left(2\right)}\leq \ldots \leq p_{\left(M\right)}.$$
- Determine the Benjamini–Hochberg critical measure for the test and find the largest *j* such that $p_{\left(j\right)}\leq \frac{j}{M}\alpha .$

- Reject all hypothesis results where $p_{\left(i\right)}\geq p_{\left(j\right)}.$

This method for correcting the correlation significance is provided in the online application. The significance test is performed using the corresponding *q*-value, which is calculated using Equation 13:

$$q_{\left(j\right)}=\frac{Mp_{\left(j\right)}}{j}$$ (13)

These calculations use the Benjamini–Hochberg FDR function in MATLAB presented by Groppe (2026). In all analyses, we used the *q*-value as a significance test, where *q* is calculated for the number of resampling instances in the Monte-Carlo simulation (as a proxy for multiple features) and for the number of correlation pairs in data analyses on the example set as well as the SIDCO+ site.

### 2.3 Network characteristics calculation

The degree of a node is defined as the number of edges connected to that node. In an undirected correlation-based graph, the degree is defined as the number of edges retained after the setting the *q*-value and lower CI boundary threshold significance value determined by the user (equal to 0.01 and 0.1, respectively). This calculation was performed using an in-house MATLAB script provided at https://github.com/computationalmetabolomicsca/sidco_plus/ based on Equation 14:

$$d_{i}=\sum_{j=1,j\neq i}^{M}k_{ij},k_{ij}=\left\{\begin{array}{c} 1,{\;R}_{n}^{*}\left(i,j\right)>0\wedge q_{ij}\leq alpha\wedge {minCI}_{ij}>{CI}^{threshold}\\ 0,{\;R}_{n}^{*}\left(i,j\right)=0\vee q_{ij}\leq alpha\vee {\min CI}_{ij}>{CI}^{threshold} \end{array}\right.,$$ (14)

where $d_{i}$ is the degree of node *i*; $k_{ij}$ is the thresholded edge between nodes *i* and *j* for an *M*-dimensional network.

Betweenness is defined as a measure of the frequency with which a node is included in the shortest path between other nodes in the graph. It is a general measure of centrality, where nodes with higher betweenness levels hold more significance in the network. This calculation was performed using a MATLAB library function with the formula given by Equation 15:

$$b_{i}=\sum_{j,t=1,j,t\neq i}^{M}\frac{s_{jt}^{i}}{s_{jt}},$$ (15)

where $b_{i}$ is the betweenness of node *i*, $s_{jt}^{i}$ is the path between nodes *s* and *t* that passes through node *i*, and $s_{jt}$ is the total number of paths between nodes *j* and *t*.

### 2.4 Monte-Carlo simulation dataset analysis

The *in silico* datasets were created using a generative modeling framework where the features were sampled from a normal distribution and measured over *n* samples. The nomenclature used here is as follows: the features are represented as *X* and *Y* with values across *n* samples, so that $X=\left\{x_{1},\ldots ,x_{n}\right\}\text{ and }Y=\left\{y_{1},\ldots ,y_{n}\right\}$ ; $\left(\begin{array}{l} X\\ Y \end{array}\right)∼ℵ\left(\mu ,∁\right)$ , with mean values $\mu^{\left(X\right)}$ and $\mu^{\left(Y\right)}$ for *X* and *Y*, respectively. The variable *X* is selected randomly, while *Y* is defined as a function of *X* along with random noise. The random numbers were generated using the Mersenne Twister method in MATLAB and included different types and levels of functional relationships. Four different groups of datasets were created, where the variable Y = f(X) followed a linear, quadratic, cubic, or sinusoid functional relationship at varying levels of random noise. These functional relationships were selected to represent possible cases of molecular dependencies ranging from linear to more complex. The period length of the Mersenne Twister method is 219,937, which allows more than 10^6,000^ draws from a sequence before repetition. The values of *Y* are calculated from *X*, as shown in Equation 16:

$$\begin{array}{l} •\;Linear\;functionality:Y=\left(aX+b\right)*Noise.\\ •\;Quadratic\;functionality:Y=\left(aX^{2}+bX+c\right)*Noise.\\ \begin{array}{l} •\;Cubic\;functionality:Y=\left(aX^{3}+bX^{2}+cX+d\right)*Noise.\\ •\;Trigonometric\;sinusoidal\;functionality:Y=\sin\left(A+BX\right)*Noise. \end{array} \end{array}$$ (16)

The noise level multiplier in the Monte-Carlo test shown here takes four different values, which represent different correlation levels ranging from strong to weak/random.

### 2.5 Experimental dataset and use-case example

The effect of changing the sample size was tested on previously published AD plasma metabolome data (Kalecký et al., 2022). Briefly, the dataset consists of targeted mass spectrometry analyses of 630 compounds, including metabolites and lipids, using ultrahigh-performance liquid chromatography tandem mass spectrometry and flow injection analysis tandem mass spectrometry with a metabolomic kit (Biocrates MxP® Quant 500). The measurements show the plasma sample profiles of 94 AD and 64 control subjects who were a part of the Texas Alzheimer’s Research and Care Consortium. All subjects were over the age of 55 years, and the sample set had the same numbers of male and female patients in each group. The demographic information of the participants and all related experimental details are provided in the original publication, which also includes extensive testing and analysis results for this dataset with particular focus on feature selection. The publicly available dataset contains a subset of 529 features for metabolites and lipids across all subjects. In our analysis, we explored the measurements provided by the authors for 87 metabolites.

### 2.6 Software availability

The open-use and open-source software was written in MATLAB backend (compiled in MATLAB 2025b) with RShiny frontend. The MATLAB code is available at https://github.com/computationalmetabolomicsca/sidco_plus. The correlation calculations were based on previously published approaches for unbiased distance correlation (Equation 6), CI using the Bernstein empirical formula (Equation 9), *p*-value using the chi-squared test (Equation 11), FDR correction for the correlation *p*-value based on the Benjamini and Hochberg (1995) procedure (Equation 13), distance correlation as a mean of the sample bootstrap where the user has the option to select the number of samples in each permutation and the number of permutations. Here, the user can choose to impute (with the K-nearest neighbor method) and normalize (using the z-score method) data in SIDCO+ or to provide data that is already normalized and does not have any missing values. The validation of these methods in terms of correlation significance for different models is demonstrated below.

**FIGURE 1:**
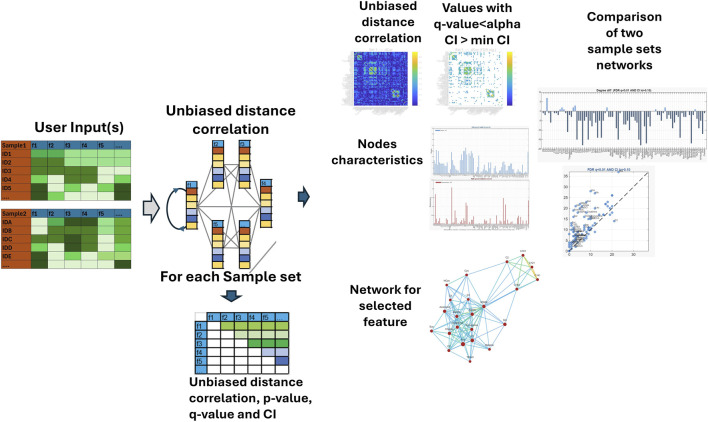
Outline of the unbiased distance correlation calculation software SIDCO+ with algorithmic solutions presented here as well as several graphical representations of the unbiased distance correlation network in one or two different datasets. The user enters one or two input data files and selects their significance levels and permutation scale. The output file provided includes the unbiased distance correlation, *p*-value (Equation 11), *q*-value (Equation 13), and minimum/maximum values of the confidence interval (CI) (Equation 9). The edges are removed if their values are below the thresholds for the *q*-value and CI entered by the user. For each input, the user can visualize plots of the node degree (Equation 14) and betweenness (Equation 15) and the graphical representation of a network for a selected metabolite. The correlation is calculated with permutations with replacement of samples (for user-selected numbers of samples and permutations) to reduce the influence of specific sample variances and allow better comparison of samples of different sizes. The software application is available at https://www.insilicobiology.ca/shiny/sidco+/.

SIDCO+ also provides graphical representations of the correlation matrix, node degree, and betweenness along with visualization of the network for user-selected features. If two sample groups are entered, SIDCO+ additionally presents graphical information about the degree and betweenness differences between these networks. All MATLAB functions used for the distance correlation calculations are posted on GitHub, and the application is available at https://www.insilicobiology.ca/shiny/sidco+/. Further information about the software, including a detailed user manual and examples of the input data and results, are available on the website. The outline of the software application is shown in Figure 1.

## 3 Results

Correlation measures the statistical association or dependence and not causality among the features in a multivariate and possibly multiomics dataset. For biological molecules, correlations can result from several processes, including enzymatic reactions, signaling, transportation, inhibition/activation etc*.* This rich source of codependence cannot be assumed to be linear or monotonic and is most likely to follow some complex functional relationship (Lo-Thong-Viramoutou et al., 2022). In contrast to Pearson correlation, distance correlation provides a measure of the non-linear relationships; unlike both Pearson and Spearman correlation methods, it can handle polytonic relationships (Székely and Rizzo, 2013a; 2013b; Monti et al., 2023). Correlation analyses for samples of different sizes require sample-size- and correlation-method-appropriate formulation of the power, deviation of correlation from the population level, and statistical significance measure. Below, we showcase the significance of the sample size problem as well as the proposal for the significance analyses with the simulated and experimental datasets.

### 3.1 Sample size has a major effect on correlation value: Monte-Carlo simulations

**FIGURE 2:**
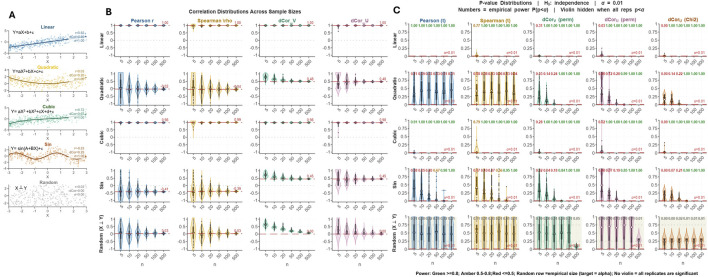
Selection of 200 random subsets for each of the six different sample sizes from 1,000 simulated points, where the correlation values are calculated using four different methods. The examples show these relationships at specific, mid/high noise levels in the Monte-Carlo simulations (provided functions). **(A)** Simulated functional dependencies used in the analyses. **(B)** Correlation levels at different sample sizes (n) and dependence types. **(C)** Obtained *p*-values using appropriate methods for each correlation type. For the unbiased distance correlation, we show the *p*-values calculated using both permutation and chi-squared tests. The significance level is assessed for *p*-value < 0.05. The corresponding plots for the low and high noise levels are shown in Supplementary Figures.

We investigated the effects of sample size on pairwise correlations. Here, we first examine two variables with simulated codependence. The datasets were created using a generative modeling framework, where the features were sampled from a normal distribution and measured over *n* samples. The nomenclature used here is the same as that introduced in the Materials and Methods section. The mean values were scaled to zero, and the variance-covariance matrix of correlations between X and Y is given by $∁=\left(\begin{array}{l} Cor\left(X,X\right)\\ Cor\left(X,Y\right) \end{array}\begin{array}{l} Cor\left(X,Y\right)\\ Cor\left(Y,Y\right) \end{array}\right)$ . Variable *X* was selected randomly, and *Y* was defined as a function of *X* at different random noise levels. In this work, we propose utilization of the unbiased distance correlation as a preferred measure in metabolomics and general omics applications by showing comparisons between the unbiased and biased distance metrics as well as the Pearson and Spearman correlations. Accordingly, four different groups of datasets were created with two variables, where the first variable was generated randomly while the second followed a functional relationship with varying random noise levels: linear, quadratic, cubic, and sinusoid. The specific functions and associated routines are provided in the Material and Methods section. These functional relationships were selected to represent possible cases of molecular dependencies, which range from linear to more complex relationships. The schematic graphs presented in Figure 2 show the different types of functional relationships (Equation 16) for the covariances used in these simulations as well as the resulting correlations for 200 replicates. *X* and *Y* were simulated for 1,000 samples each, and the correlations for different sample sizes were calculated through random sampling with replacement from this set.

The Pearson, Spearman, biased distance, and unbiased distance correlation values for the *X* and *Y* pairs in these sets are shown in Figure 2 along with their corresponding *p*-values. The calculations shown in Figure 2 were performed on the Monte-Carlo simulations with 1,000 *Y* data points being determined from randomly generated *X* values with different functional dependencies at specific noise levels. The correlations were calculated at different sample sizes and selected randomly with replacement from the full set 200 times for each sample size. The equivalent plots for noise levels of 0.1 and 10 are shown in the Supplementary Material; the MATLAB code used to generate these figures is available in GitHub.

As expected, both biased and unbiased distance correlations are excellent for identifying complex functional dependencies and show effective information about the correlations in the linear, quadratic, cubic, and trigonometric functions considered here. In contrast, the Pearson and Spearman correlation methods fail to detect non-linear polytonic relationships. All methods show sample-size and correlation-strength-dependent value ranges. Here, biased distance correlation has the largest sample-size dependence, and the bootstrap mean values for the low and medium correlation levels deviate significantly from the population levels. The results shown in Figure 2 and the Supplementary Material indicate that the average correlation levels are higher in smaller sample sizes, with the most significant errors occurring in weak and medium correlation groups and in biased distance correlation. In strongly correlated pairs, the correlations remain strong at small sample sizes. The *p*-values decrease with increasing sample sizes; this effect is more pronounced in weaker correlations, where significant *p*-values are only achieved at larger sample sizes. For *p*-values obtained with the permutation tests in both distance correlation formulas, the majority of correlations at the small sample size (five samples) fail the significance test for values below the strongest level. The limited possibility of perturbation at this low sample size renders the results uncertain.

**FIGURE 3:**
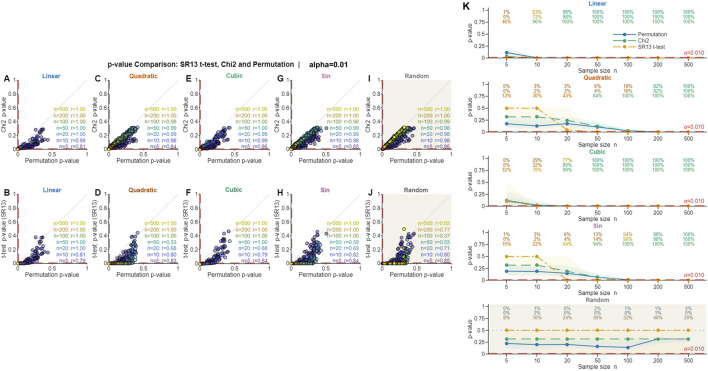
Added noise (multiplier = 1) leads to intermediately strong correlations (equivalent plots for multiplier levels 0.1 and 10 are shown in the Supplementary Material). In all plots, the x-axis corresponds to *p*-values obtained using the permutation test with 500 permutations. **(A, C, E, G, I)** The y-axis shows values obtained using the chi-squared method (Equation 11) based on Shen et al. (2022); **(B, D, F, H, J)** the y-axis shows *p*-values obtained using the formula in Equation 12 for the limit of high dimensionality presented by Székely and Rizzo (2013a) and Gao et al. (2021). The plots include Pearson correlation values between the *p*-values obtained with the two methods compared in the plots (r) as well as the corresponding sample sizes (n). **(K)** Changes in *p*-values for the three methods based on sample size for different functional relationships; the corresponding plots for different noise levels are shown in the Supplementary Material.

**FIGURE 4:**
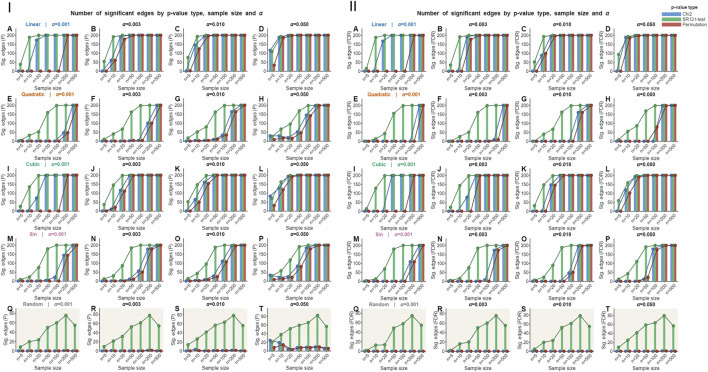
Number of correlation pairs that pass different significant level points based on three different p-value calculation methods. I p-value is used directly as is; II p-value is corrected using FDR.

For unbiased distance correlation, Székely and Rizzo (2013a) and later Shen et al. (2022) showed that the chi-squared test provides comparable *p*-values to the permutation test for sample sizes over ∼20. We confirm these findings here, and Figure 3 shows the comparisons for *p*-values obtained using the chi-squared method of Shen et al. (2022) and permutation test (Figure 3I); the results show that the values agree in the significant range for all models and that the overall correlation of values is very high even in sets with only five samples, with perfect correlations being obtained for 10 or more samples. In addition, we show comparisons between the *p*-values obtained using the permutation test and t-test methods of Székely and Rizzo (2013b) and Gao et al. (2021). While the chi-squared method shows excellent agreement with values of the permutation test, there are differences with the *p*-values obtained using the t-test method of Szekely and Rizzo (2013a) (SR13 method) as well as permutation tests with less than 50 samples, particularly in the random group. In our low-dimensional pairwise simulations, the high-dimensional t-test approximation showed poorer agreement with the permutation *p*-values than the chi-squared approach. However, the chi-squared test has been previously proven (Shen et al., 2022) to be an excellent substitute for the permutation test, especially with its faster calculation time. The *p*-value dependence on sample size (Figure 3K) and power and type I error analysis results (Supplementary Material) show further agreement between the permutation and chi-squared *p*-value tests. For these same examples, Figure 4 shows the numbers of significant values selected for three different *p*-value tests at different alpha values and sample sizes at noise level 1; the results for noise levels 0.1 and 10 are shown in the Supplementary Material.

At alpha = 0.003, the chi-squared and permutation *p*-values show full agreement in all cases for all sample sizes tested in the case of significance levels determined from *p*-values as well as following FDR reevaluations. This is also true for the three different noise levels simulated herein (Supplementary Material). For the SR13 method, the *p*-values show agreement for very high correlation levels, while a large number of samples at a low correlation limit generally result in much lower *p*-values than the other two methods. This observation is in agreement with the assumption that this method requires a sample size approaching infinity. Additional empirical power and type I error analyses results are shown in the Supplementary Material for these functional dependency simulation pairs, confirming that the permutation and chi-squared *p*-values agree in all cases tested, while the SR13 method has very high type I error rates at small sample sizes. Figure 3K and the type I error analysis results (Supplementary Material) show that alpha = 0.01 (or 0.003 for a more stringent analysis) leads to extremely low type I errors with n = 500 samples while retaining significance for even n = 5 samples in correlated pairs. For further analyses and in the SIDCO+ application, we use the chi-squared method of Equation 11 reported by Shen et al. (2022) as it provides comparable results to the permutation test with significantly faster calculations and no dependence on the number of permutations.

### 3.2 CI and deviation from population level in unbiased distance correlation

The sampling errors for properties following U-statistics, such as the unbiased distance correlation (Ai et al., 2023), and CI derivations for U-statistics (Nguyen, 2019) are noted above in the form of Equation 9 for general cases. The alternative CI measures include Bayesian bootstrap analysis or Fisher-transformation-based error bounds (see Materials and Methods).

**FIGURE 5:**
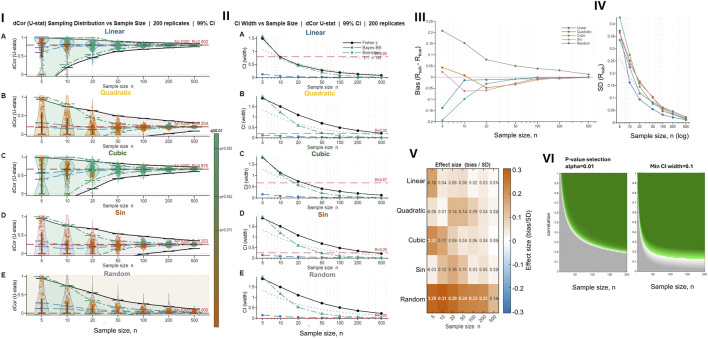
Analysis of CI using three different methods for Monte-Carlo simulations of the distance correlation values. **I**. Panels show values for 200 iterations with replacement at different sample sizes. The values in green exceed the statistical significance threshold (0.01), and the lines for the CI boundaries are indicated for the Fisher (black), Bayes bootstrap (blue), and Bernstein (green) methods. **II**. Upper CI boundaries of the three methods. **III**. Biases of the correlation values for a sample size *n* compared to the sample size *N* = 1,000 of the complete set. The bias values are shown for different functional dependencies, including random values. **IV**. Standard deviations (SDs) of the dCor values obtained at different sample sizes for the functional dependencies tested. **V**. Effect size comparison for different sample sizes against *N* = 1,000, calculated as Cohen’s d-average ratio of the bias and mean SD (Lakens, 2013). **VI**. For a *p*-value threshold of 0.01 and minimum CI threshold of 0.1, the correlation values that exceed the threshold (green) at different sample sizes are shown. The example shows that for most of the sample sizes, the *p*-value and CI thresholds provide equivalent correlation level significance, while a lower CI boundary threshold adds further stringency at small sample sizes.

Figure 5 shows the analysis of CI methods and bounds using the proposed approaches for a noise level of 1 (equivalent analyses for noise levels of 0.1 and 10 are provided in the Supplementary Material). The Fisher bounds have the widest range and thus include all values but provide very broad limits. The Bayesian bootstrap method only corresponds directly to values that were obtained with the bootstrap calculations, thus providing a narrow range for the sampled values. The Bernstein method provides the best range coverage at low sample sizes, with the range being dependent on sample size; in the examples shown, the ranges also include the results of a large sample size for all *n* values.

We also explored the bias, standard deviation (SD), and effect size (Figure 5III, IV and Supplementary Material) to demonstrate the differences with unbiased distance correlation for a given sample size. The bias is calculated as the difference between the mean of the unbiased distance correlation obtained for a specific sample size over 200 permutations and the value for the complete set (1,000 samples). Figure 5III shows the bias values for different functional types at different sample sizes. The bias clearly approaches zero at larger sample sizes, with the difference being very low even at *n* = 20 in these cases. The mean value of the bootstrap correlation analysis thus provides a method for determining the population-level correlation value based on this empirical analysis. The statistical significance of the difference at *n*-samples to values at *N* is indicated when present (or absent in all cases); unsurprisingly, the largest difference is observed for the smallest sample size and for fully random *Y*, i.e., the weakest correlation level. Similar results are observed for noise levels of 0.1 and 10 (Supplementary Material). The effect size is calculated here as the Cohen’s average d-value given by

$$d_{av}=\frac{\left|\bar{{dCor}^{*}\left(n\right)}-\bar{{dCor}^{*}\left(N\right)}\right|}{\left({SD}_{n}+{SD}_{N}\right)/2}.$$

For all values shown here, including the functional dependencies at noise levels of 1 (Figure 5), 0.1, and 10 (Supplementary Material), $d_{av}$ is very low for all cases and indicates indistinguishable results for smaller sample sizes within these boundaries and with permutation analysis. Only the random values for *Y* where the distance correlation approaches zero show some differences in effect size; this suggests exclusion of low values based on the Bernstein lower CI bound as necessary.

We propose that the unbiased distance correlation estimations can be calculated as means of the bootstrapped values; further, the significance for any sample size can be considered based on the *p*-value and CI calculation in the range of $\frac{2\log\left(2/\delta \right)}{3\left(n\right)}$ . The significant correlations can then be selected using combined significance based on the chi-squared *p*-values with FDR correction and CI range, where choosing only values with the minimum CI exceeding the user-defined level ensures selection of significant pairwise correlations at sample-size-appropriate levels (an example of significance achieved for *p*-value and minimum CI is shown in Figure 5VI). Excluding edges with minimum CI below the threshold ensures that only very strong correlation values are retained as significant at low sample sizes, while selecting a narrow CI ensures that even weak correlation values are retained for large sample sizes. As an example, based on the combined threshold selection approach, a correlation of 0.2 will be statistically significant only at a sample size of ∼200 (Figure 5VI). This approach will be further demonstrated in an experimental example. The methodology is publicly available as part of the SIDCO+ application.

### 3.3 Application example: AD metabolomics dataset analysis

The utility of unbiased distance correlation with *p*-values and the CI is explored for a previously published AD plasma metabolome dataset (Kalecký et al., 2022). Briefly, the dataset consists of measurements from 94 AD and 64 control subjects; all subjects were over the age of 55 years, and the sample set had the same numbers of male and female patients in each group. The demographic information of the participants and all experimental details are provided in the original publication. The panel measurements discussed in the original publication included 106 metabolites and 524 lipids that were analyzed using a targeted kit (Biocrates life Sciences AG). The publicly available dataset contains a subset of 529 features, including 87 metabolites across all 158 subjects. The original publication also includes extensive testing and analysis results for this dataset, with particular focus on feature selection. In our analysis, we consider the measurements provided by the authors for the 87 metabolites as a demonstration of the proposed method. The goal of this analysis is to identify differences in metabolic correlation networks between the AD and control (NC) groups using the approaches presented herein as well as calculate the FDR-corrected *p*-value and CI. In the following analysis, all references to correlation imply the unbiased distance correlation; the analysis presented here uses methods that are publicly available in SIDCO+.

**FIGURE 6:**
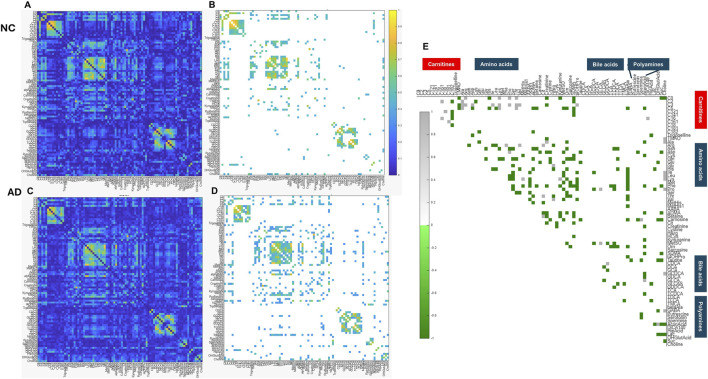
Unbiased distance correlations for the control (NC) and Alzheimer’s disease (AD) groups. All calculated correlation values for the **(A)** NC and **(C)** AD groups. Results of correlation values that do not satisfy either the *q*-value threshold of alpha = 0.01 or minimum CI of 0.1 are set to 0 (shown in white in the plot) for the **(B)** NC and **(D)** AD groups. **(E)** Difference between the NC and AD group correlations with hard thresholding; the green points indicate pairwise connections that are only present in the AD group, while the gray points indicate pairwise connections present only in the NC group. The major metabolite groups considered are indicated in the panels.

Prior to analysis, the data were imputed with the K-nearest neighbor method (K = 5), and the z-scores were normalized across features for each dataset separately (SIDCO+ allows preprocessing with imputation and normalization options). In this analysis, we selected alpha = 0.01 and allowed only correlations satisfying a lower CI boundary >0.1. The correlations were calculated with bootstrapping for 100 permutations and a random selection of 50 samples. The *p*-value and CI were calculated for the mean correlation value from the bootstrap. The initial overview of the unbiased distance correlations for the NC and AD groups is shown in Figure 6, where Figures 6A,C show directly calculated correlation values for the two groups while Figures 6B,D show statistically significant values following selection with alpha = 0.01 for the FDR-corrected *p*-values (*q*-values) and minimum CI = 0.1 for the Bernstein CI values. Figure 6E shows the difference between the AD and NC networks.

The subset of pairwise correlations shown in Figures 6B,D satisfy the strict and sample-size-dependent significance thresholds. Here, the AD group has several new edges relative to the NC group with stronger relationships between, for example, bile acids and amino acids as well as carnitine and polyamines (Figure 6E). This finding corroborates earlier results showing the significant role changes of bile acids in AD, as reviewed by Singh and Aran (2026). Succinate (Suc) and lactate (Lac) show major differences in the networks that increase the number of correlations in the AD group, while choline loses edges with amino acids in AD (Figure 6E). Although the correlation analysis does not necessarily indicate direct metabolic reactions, it shows more significant metabolic proximity in AD. For example, Suc and Lac have been previously linked to metabolism of NAD+, a major factor in the development and progression of AD (Meng et al., 2024; Andreyev et al., 2024; Chaubey et al., 2025); thus, although the focus of the present work is not on the biological interpretation of these results, it is important to note that the identified differences align with findings from other studies highlighting ametabolic changes in AD (Polis et al., 2021).

**FIGURE 7:**
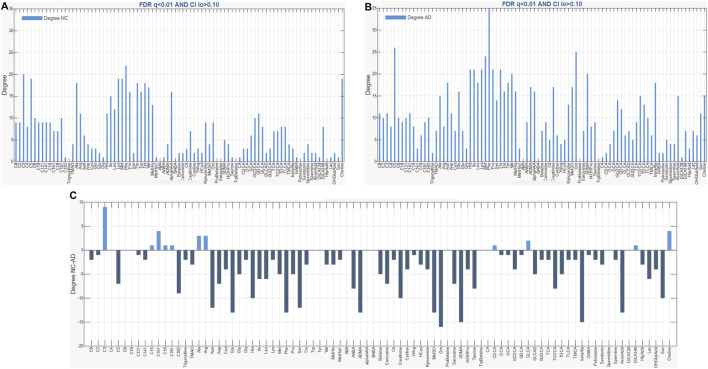
Degree of each node (i.e., metabolite) calculated as the number of statistically significant edges at each node. The statistical significance is determined in terms of both *q*-value and minimum CI. Degree of each node in the **(A)** NC and **(B)** AD groups. **(C)** Difference between the node degrees of the NC and AD sets for each metabolite.

**FIGURE 8:**
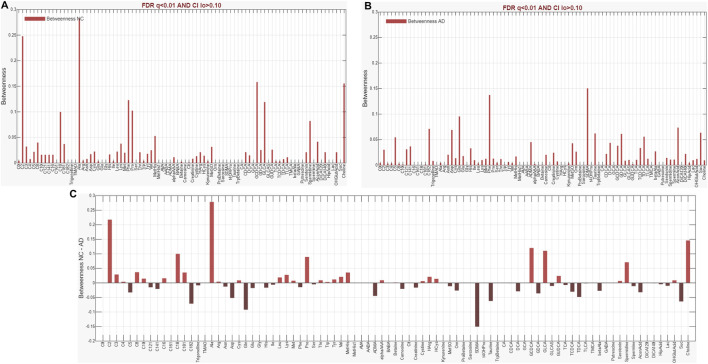
Betweenness of each node (i.e., metabolite) measured as the fraction of shortest paths between all pairs of other nodes that pass through the defined node for the sum of these fractions across all node pairs. Higher scores indicate a higher capacity to act as a bridge or connector for each node. The statistical significance is determined in terms of both *q*-value and minimum CI. Betweenness of each node in the **(A)** NC and **(B)** AD groups **(C)** Difference in node betweenness for the NC and AD sets for each metabolite.

Further characteristics of the networks can be explored by examining the properties of the nodes in the network. As major characteristics, the node degree and betweenness are provided (Figures 7, 8). The node degree is calculated as a number of edges for each node (following statistical significance selection). For the majority of metabolites, the node degree increases in the AD group, as can be expected from gaining significant pairwise correlations relative to the NC group. Interestingly, phenylalanine (Phe) has the highest node degree in both the NC and AD groups, albeit with almost twice the degree level in AD owing to correlations with new partners in this group, including bile acids, amino acids, and organic acids. Overall, a much higher node degree in AD suggests a stronger and more interconnected network in the disease state than NC. The node degrees and degree difference between the two groups for each node are shown in bar graphs (Figure 7) and scatter plots (Supplementary Material), with the corresponding methods made available in SIDCO+. Thus, major increases in the number and weights of the edges are apparent in AD.

The betweenness centrality for the nodes is overall higher in the NC group; this indicates that the NC network has a more bottlenecked and centralized structure. In this case, the nodes or clusters of nodes that are otherwise separated are linked through a few critical connectors (Figure 8). Nodes with the highest betweenness are alanine (Ala) in NC and symmetric dimethylarginine (SDMA) in AD. Importantly, in both cases, these metabolites have very low betweenness in the other group. The differences in betweenness for the metabolites in these two networks are shown in Figure 8C, which clearly indicates changes in node significance between the two networks.

**FIGURE 9:**
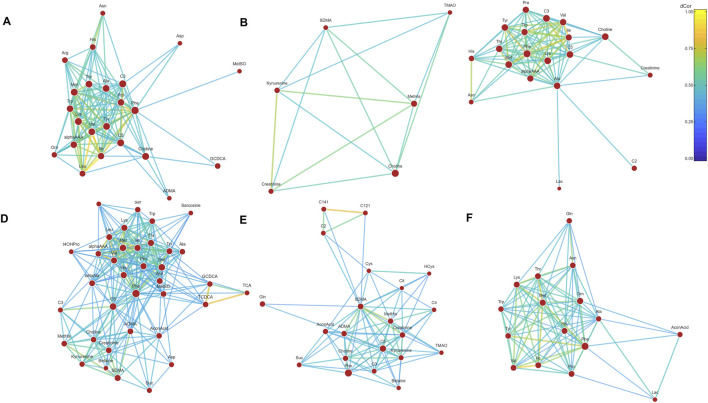
Calculated metabolic networks for selected metabolites in the **(A–C)** NC and **(D–F)** AD groups. The correlation strength is indicated by the edge color. Only the connections that pass the significance tests for phenylalanine (Phe), symmetric dimethylarginine (SDMA), and alanine (Ala) are shown.

SIDCO+ additionally provides the facility to explore specific connections for selected metabolites in the calculated network. As an example, Figure 9 shows the direct neighbors of SDMA, Phe, and Ala in the NC and AD networks; here, SDMA has the largest increase in centrality in AD, while Phe has the highest degrees in both groups with a major increase in the number of connections in AD. Figure 9 shows that SDMA is a major network hub in the AD network that links the cluster of carnitines with the cluster of amino acids and organic acids. The betweenness of Ala noted in NC is majorly reduced in AD, where it loses edges to choline, creatine, and C3.

## 4 Discussion

Accurate calculation of the relationships between biomolecules is a critical step in both data-driven and hybrid data-knowledge-driven network derivations that are often used to compare metabolic network states between different sample types. Correlation analysis is often preferred as a tool in bioinformatics, with the applications ranging from simple pairwise analysis to complex network-inference and machine-learning tasks. However, correlation measurements can be affected by sample variability and size, possibly leading to erroneous results; further, the often-utilized Pearson and Spearman correlation methods do not cover all functional relationships. These problems are particularly concerning when comparing correlation values or networks of complex datasets with different sample sizes. Novel approaches, such as methods presented by Li et al. (2024), provide interesting avenues for calculating the inverse Jacobian matrix with the aim of determining both edge weights and directionality for specific reactions in the network; however, this approach assumes the availability of many samples (∼1,000), which is a rarity in biological experiments. Bias-corrected distance correlation provides a robust method for determining the covariances between biomolecules. Our Monte-Carlo analysis illustrates the unique strength of this unbiased distance correlation in determining complex relations, with major sample size changes observed in the biased distance correlation being significantly reduced in this formulation. In the simulated dataset, we include four different functional dependencies relevant to high-throughput biological data demonstrating linear, monotonic, and polytonic relationships between the variables. Our simulations additionally include the contributions of increasing noise levels, thereby simulating varying correlation levels (Supplementary Material). We show that the chi-squared *p*-values based on the Benjamini and Hochberg (1995) FDR and Bernstein empirical inequality for U-statistics provide effective measures of significance for the bias-corrected distance correlations. In the simulated examples, the effective threshold values were alpha <0.01 and a lower CI bound of 0.1; however, different levels of stringency are possible and may be set depending on specific data and research questions.

Analysis of the simulated dataset shows that in an extremely small set of samples, the combined CI level and *p*-value thresholds minimize false positive discovery; in larger sample sets, this approach ensures that even low correlation levels (above a minimum CI threshold level) can be detected with the established error range to reduce false negative levels. In the simulated case, our approach provides a good estimate of the range of values and covers 95% of the values obtained for all sample sizes.

**FIGURE 10:**
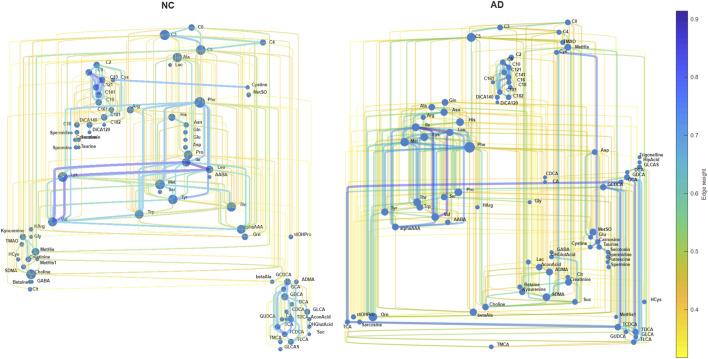
Significant unbiased distance correlation edges in the NC and AD groups obtained using the features available in SIDCO+. Here, the node size corresponds to the node degree, while the edge color corresponds to the correlation value. The correlation edges are thresholded at alpha = 0.01 and a lower CI boundary of 0.1 and calculated as the mean of 20 bootstrap permutations at a sample size of 50 for both groups.

As an illustration of the proposed approach, we demonstrate the determination of major correlation differences between the AD and NC groups using data from a previous work (Kalecký et al., 2022). Here, the dataset contains measurements for 64 control and 94 AD subjects, which makes direct comparative analysis between the two groups problematic owing to the sample size difference. Sample-size-appropriate significance determination allows comparative analyses of correlation networks between the NC and AD groups. Our analysis approach shows major changes in the node degree and betweenness in the correlation network for the number of metabolites. Overall, there are more edges in the AD than NC network, while the NC network has higher betweenness levels that indicate the presence of bridges, i.e., central nodes connecting metabolite neighborhoods. Differences in biological pathways related to the number of metabolites showing significant changes in betweenness or degree between AD and NC have been documented previously (Meng et al., 2024; Andreyev et al., 2024; Chaubey et al., 2025; Polis et al., 2021). Figure 10 shows the overall networks for the NC and AD groups obtained using the methods presented herein. Several network differences are apparent in the figure, such as the close associations of polyamines, spermidine, spermine, and putrescine with bile acids (e.g., GLCA and GLDCA) in only the AD group, as observed elsewhere recently (Li et al., 2024; Mohanty et al., 2024). Specific consideration of metabolites with major changes in degree or betweenness also refer to several previously proposed metabolic factors, including amino acids such as glutamine and phenylalanine (Figure 9 and Supplementary Material).

The AD analysis presented here is intended as an illustrative use case for the proposed network-comparison workflow. Hence, the edges and hub metabolites identified here should be interpreted as hypothesis-generating associations rather than causal or pathway-level validations, particularly because the correlation networks may reflect indirect dependencies, shared confounding, imputation effects, or group-specific variance structures. The methodologies presented herein provide a set of novel approaches for determining the range of values for calculated unbiased distance correlation. In the online application (https://www.insilicobiology.ca/shiny/sidco+/), users can obtain unbiased distance correlation values for their sample set, *p*- and *q*-values, lower and upper correlation limits, and a graphical representation of the distance correlation edges following threshold selection (detailed information about the application is provided online).

An important contribution of this work is the development of a bootstrap method for calculating the unbiased distance correlation values and determining their CI range, which enables utilization of the calculated values with awareness of the sample size effects. Our method allows more accurate comparisons of the correlation values between samples of different sizes through bootstrapping and the selection of significant values based on both the *q*-value and CI. Furthermore, we tested the chi-squared *p*-value methodology established previously and enable its use in this application. Future works will explore the application of this correction approach in the calculation of the inverse Jacobian matrix as well as the application of other selection approaches, including proportional thresholding (retention of some percentage of the strongest edges), graphical lasso (glasso), and other methods reviewed recently (Masuda et al., 2023), to determine the network using unbiased distance correlation values.

Correlation analysis continues to be a cornerstone of computational biology investigations and is often used to compare molecular interactions across different sample sets. The present study highlights that differences in sample size can influence the obtained conclusions because of correlation dependence on the sample size. We show the advantages of unbiased distance correlation in the analysis of unknown types of relationships but also highlight possible issues caused by the sample-size-dependence of the calculated correlations. Using formulas derived from sampling theory, we present a method for determining significant correlation edges in sample sets of varying sizes. In addition, we showcase the application of this approach for comparing sample sets, which provides a notable improvement in the accuracy of the estimated correlation values.

## Data Availability

Publicly available datasets were analyzed in this study. These data can be found here: https://doi.org/10.3233/jad-215448.
