# Supplementary figures and images for: Unbiased distance correlation with sample-size-aware confidence bounds for comparative omics network analysis
Source: Front Bioinform. 2026 Jun 11;6:1788010. doi: 10.3389/fbinf.2026.1788010 (PMC13294210; doi:10.3389/fbinf.2026.1788010)

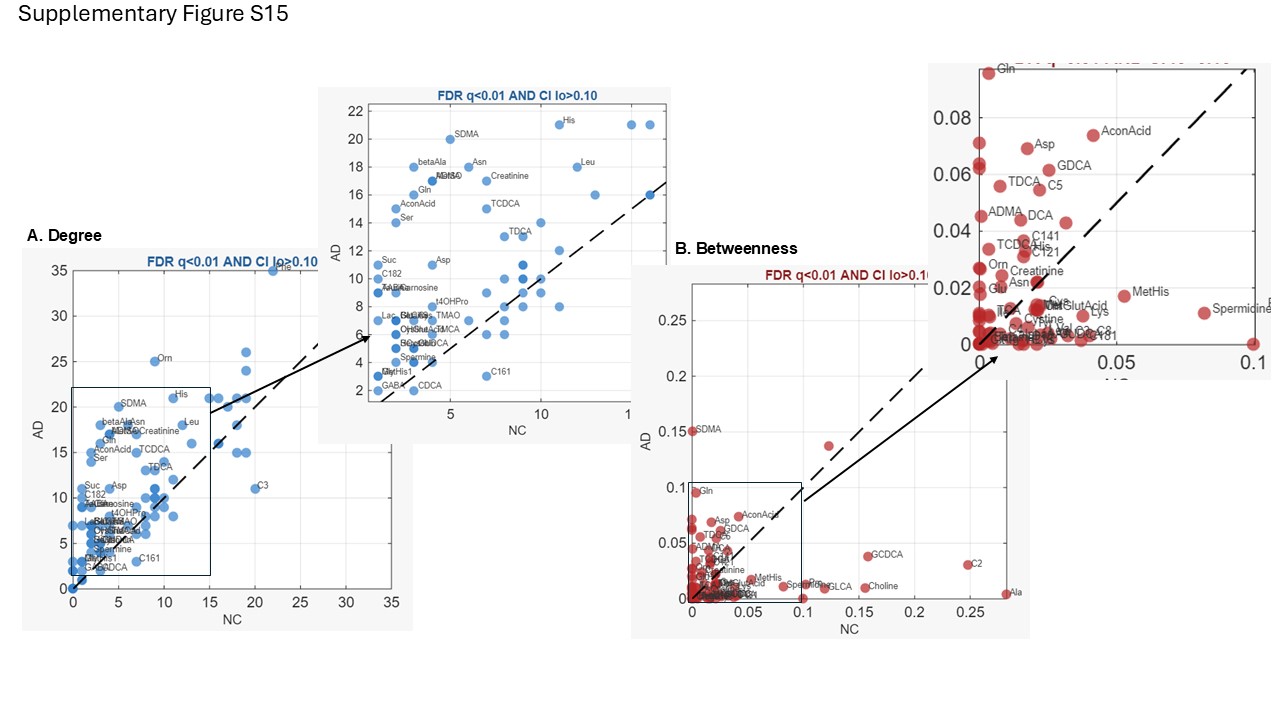

Supplement: Supplementary file 1 [file Image15.jpeg]

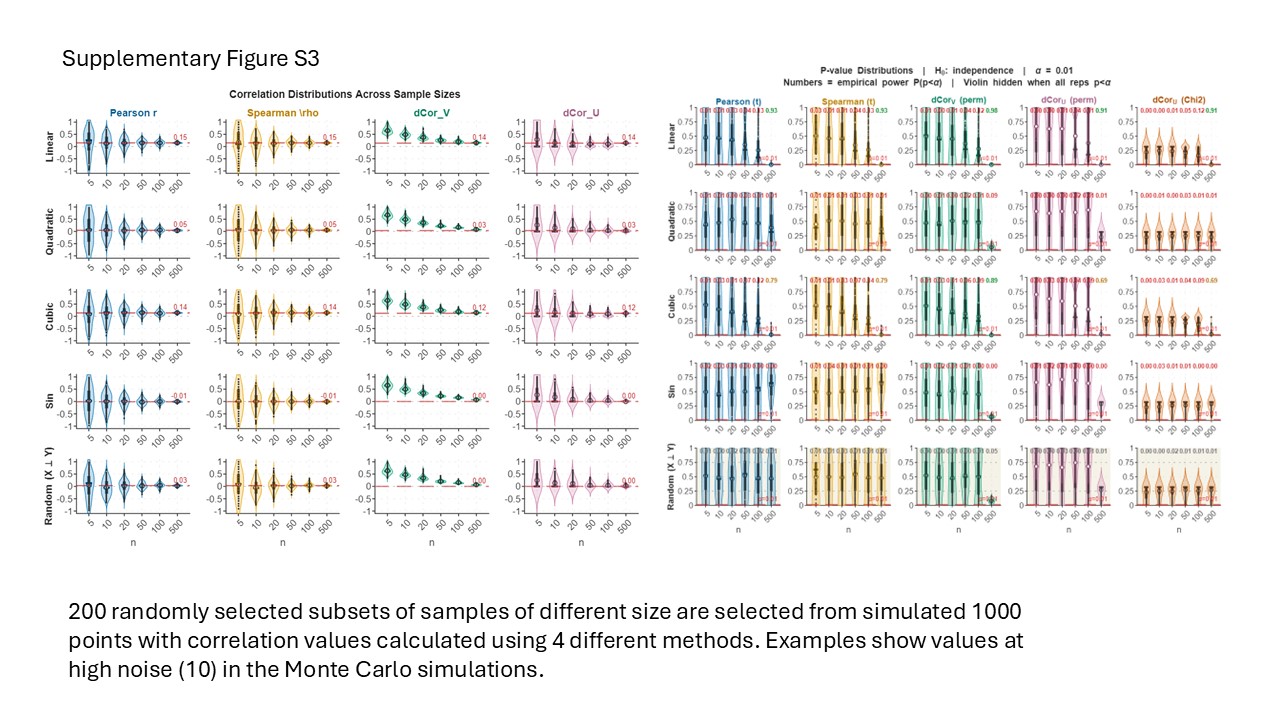

Supplement: Supplementary file 2 [file Image3.jpeg]

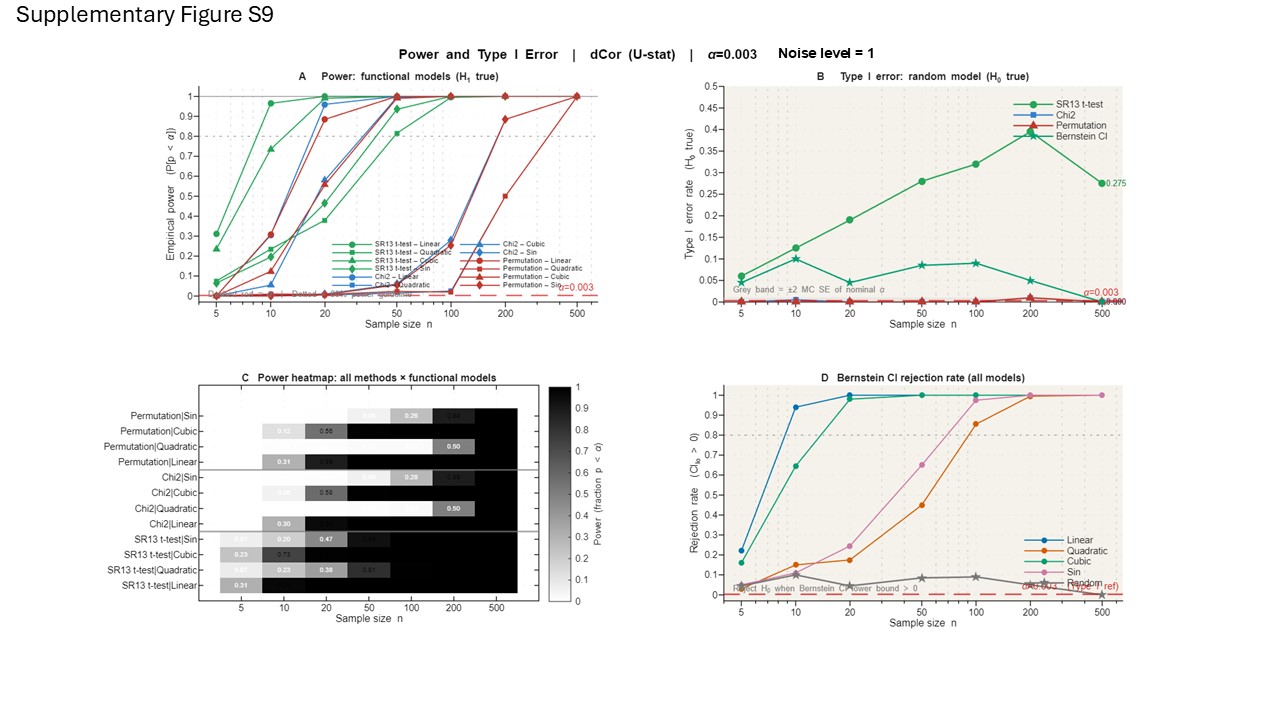

Supplement: Supplementary file 4 [file Image9.jpeg]

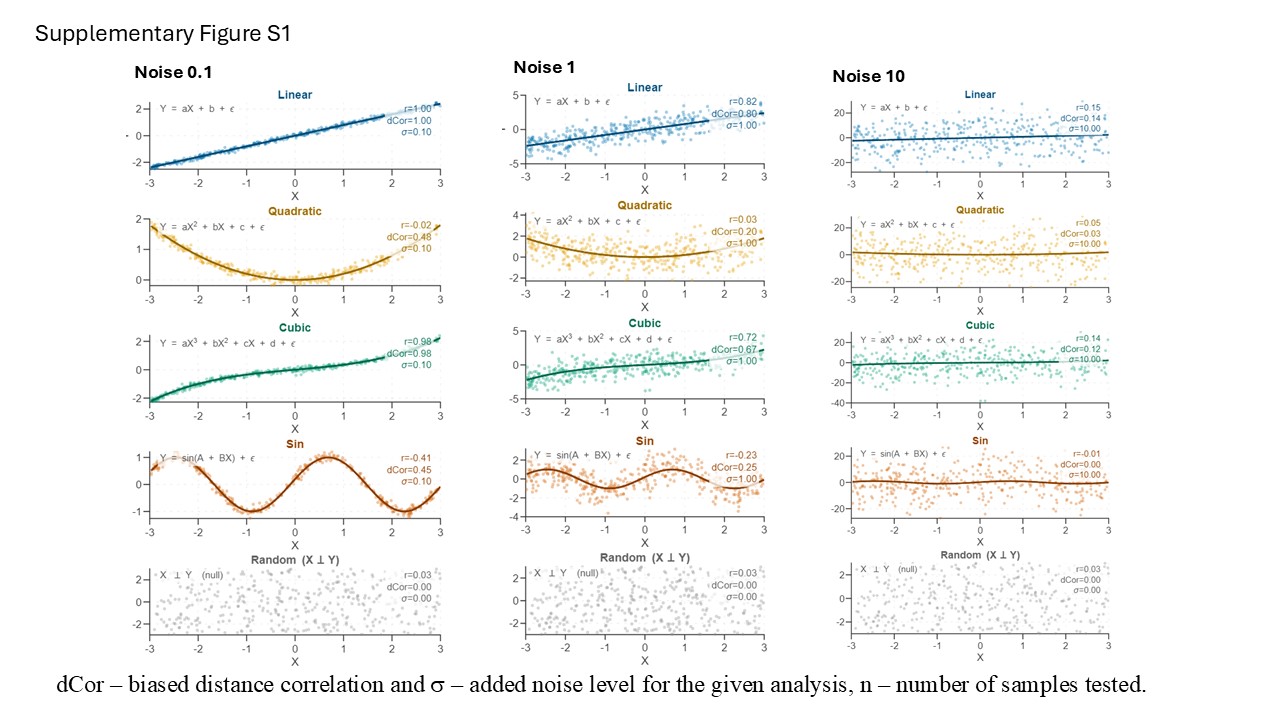

Supplement: Supplementary file 5 [file Image1.jpeg]

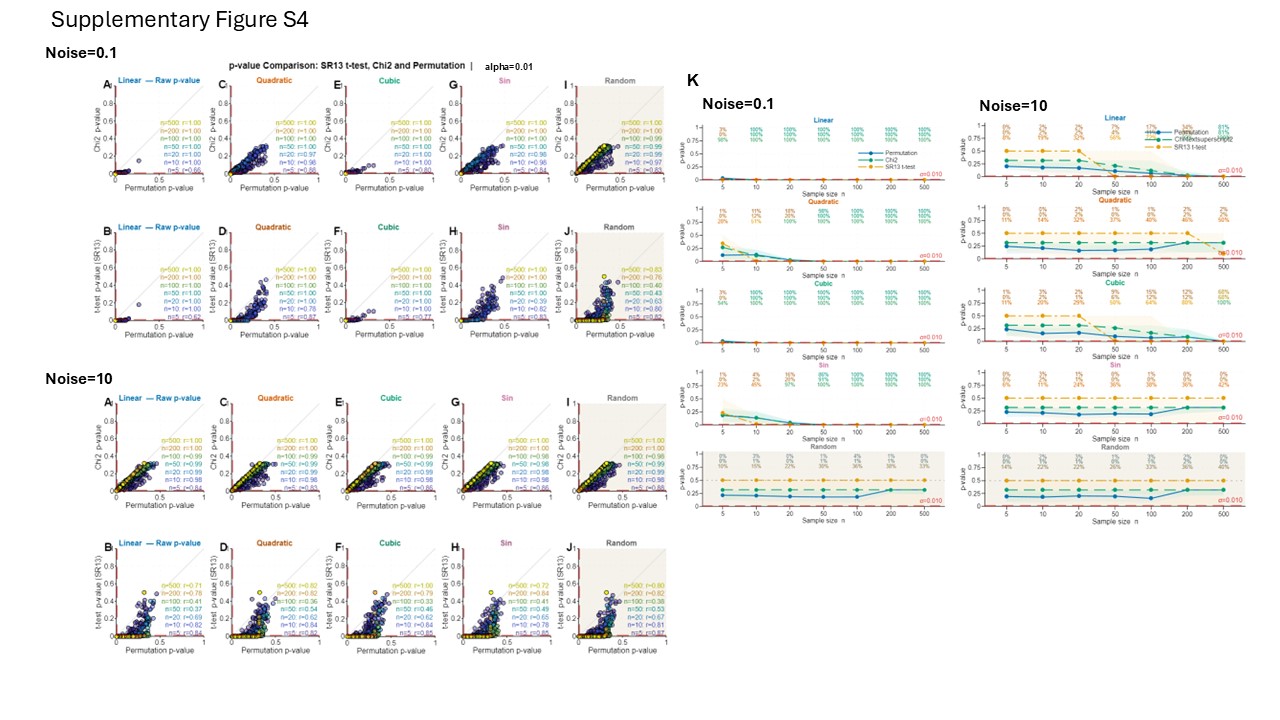

Supplement: Supplementary file 6 [file Image4.jpeg]

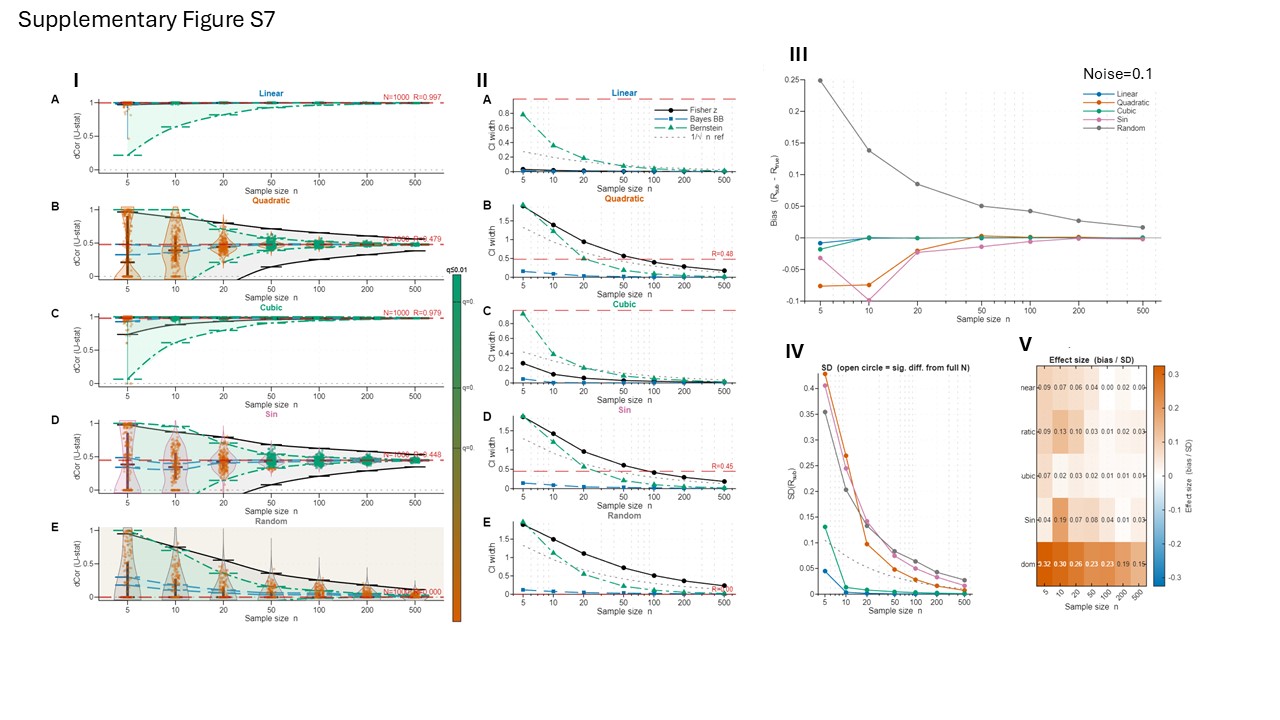

Supplement: Supplementary file 7 [file Image7.jpeg]

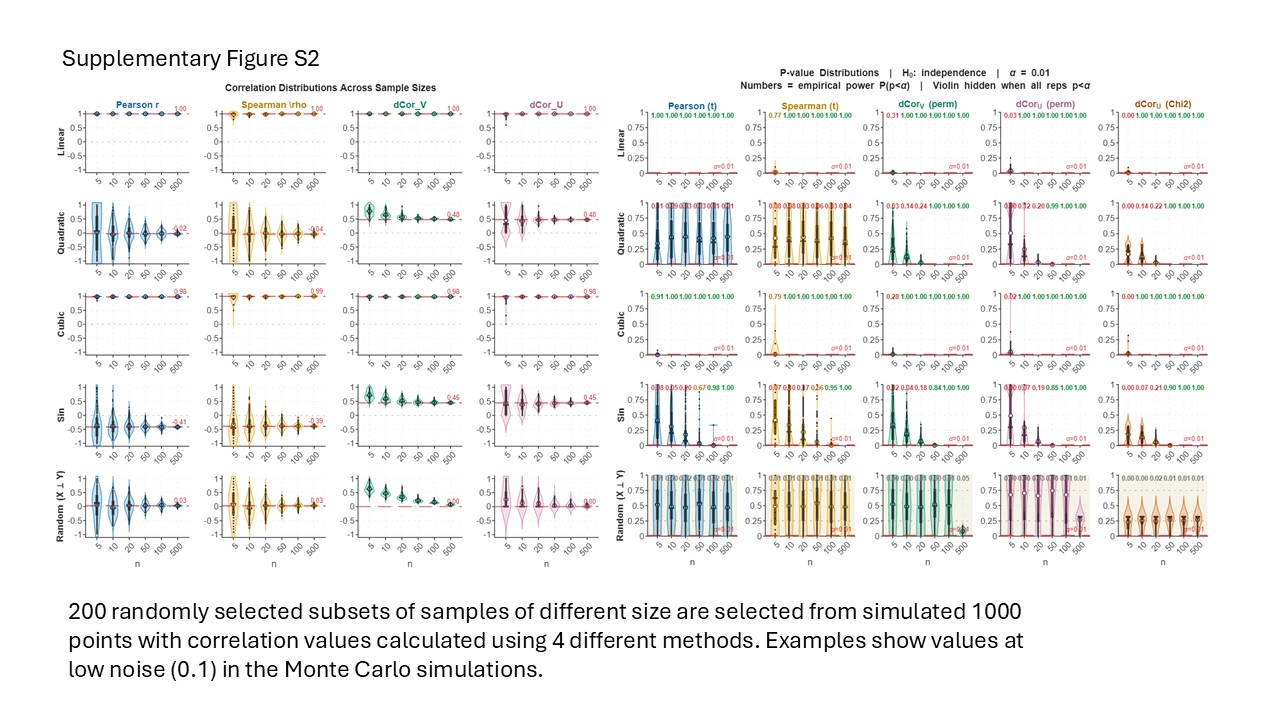

Supplement: Supplementary file 8 [file Image2.jpeg]

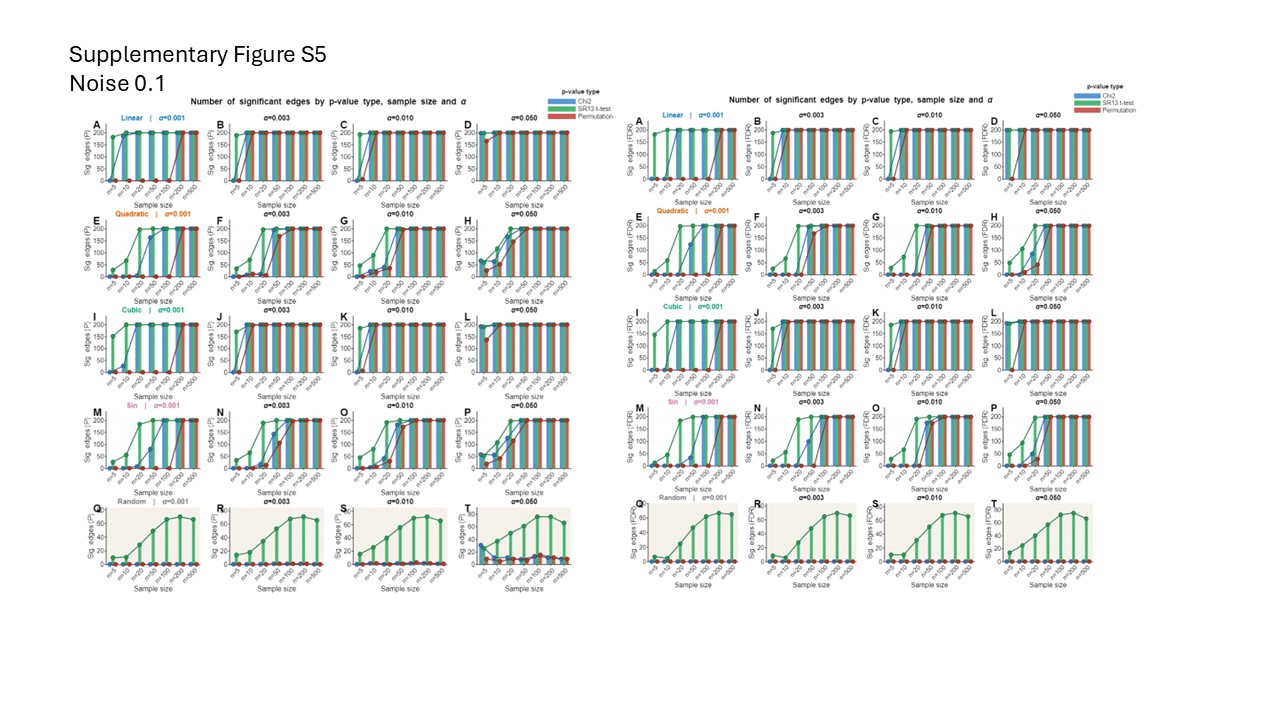

Supplement: Supplementary file 9 [file Image5.jpeg]

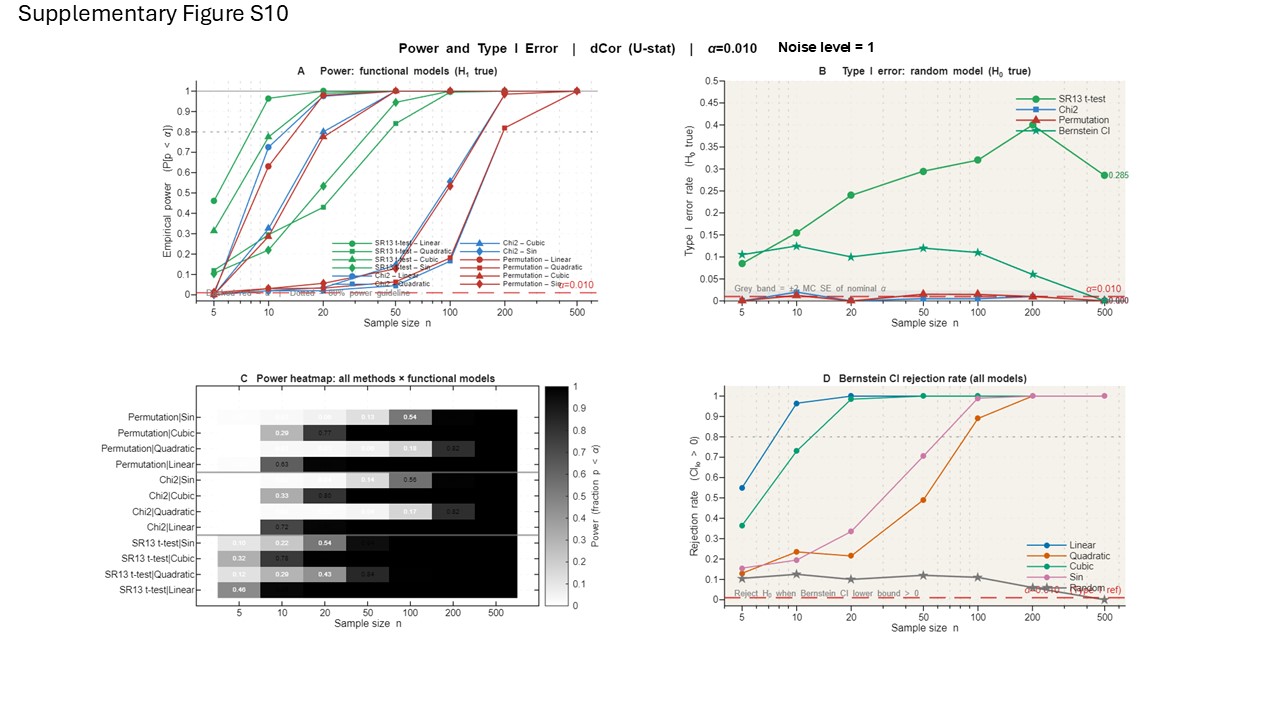

Supplement: Supplementary file 10 [file Image10.jpeg]

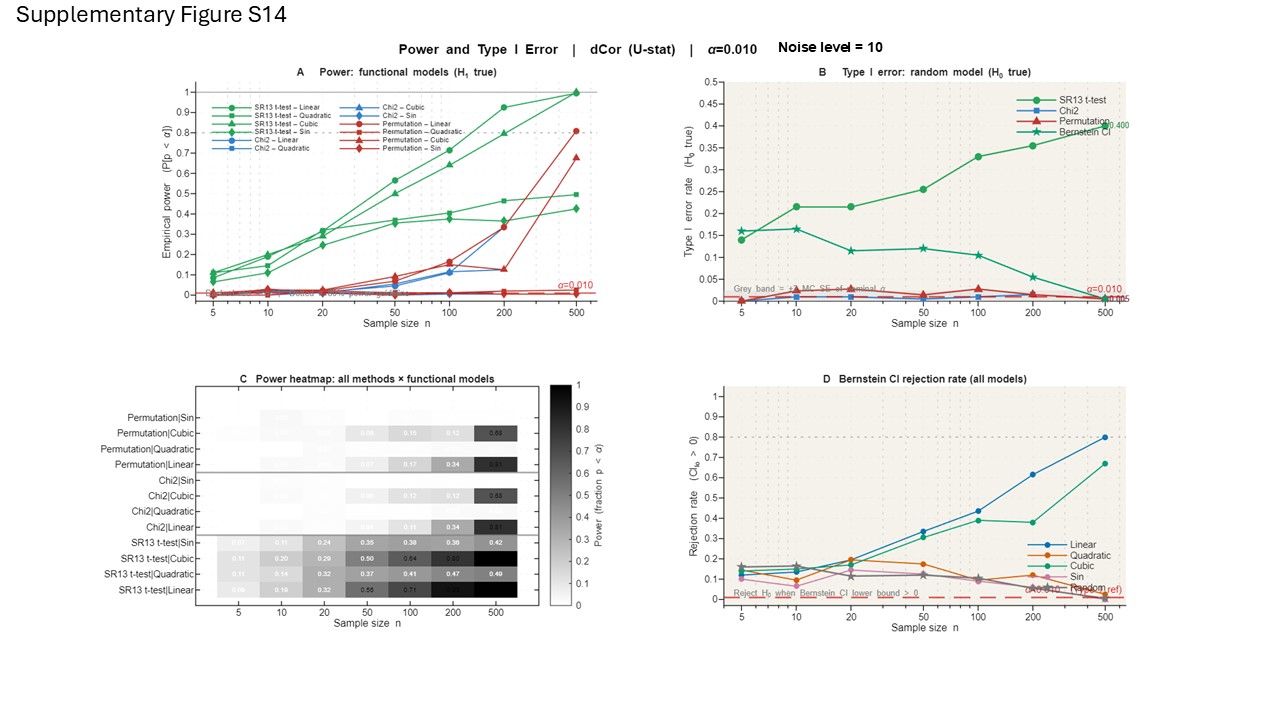

Supplement: Supplementary file 11 [file Image14.jpeg]

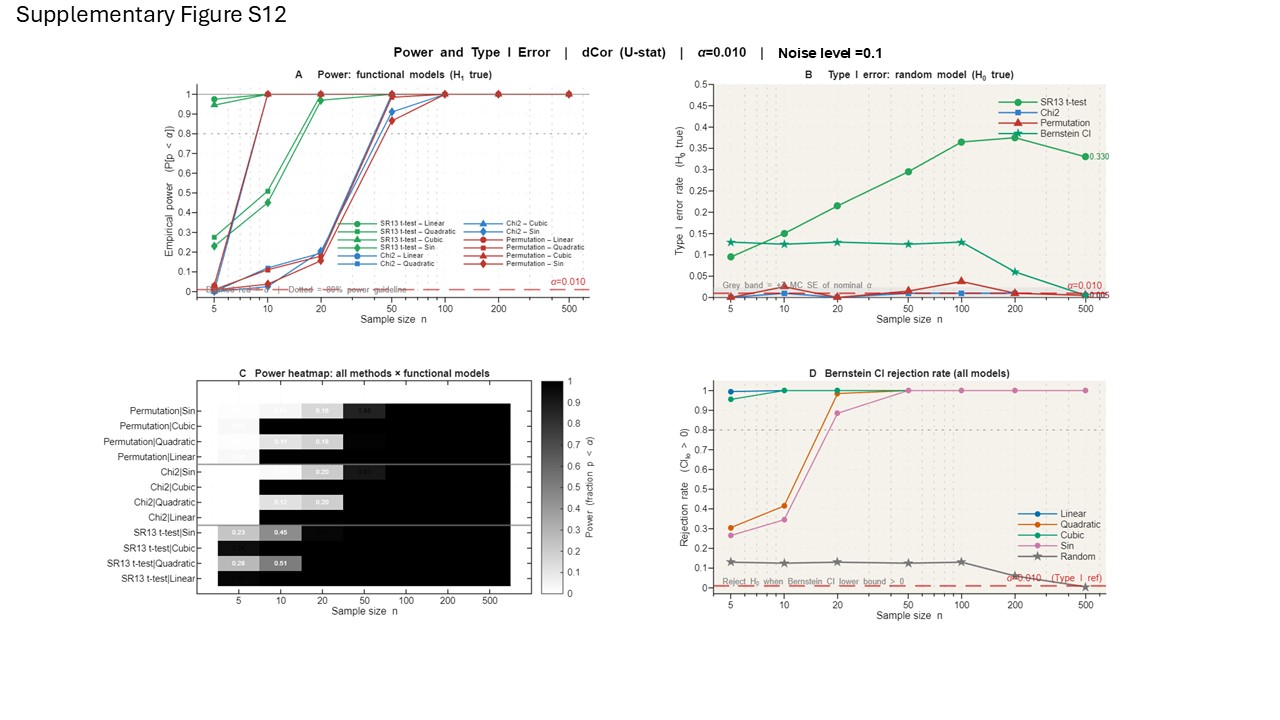

Supplement: Supplementary file 12 [file Image12.jpeg]

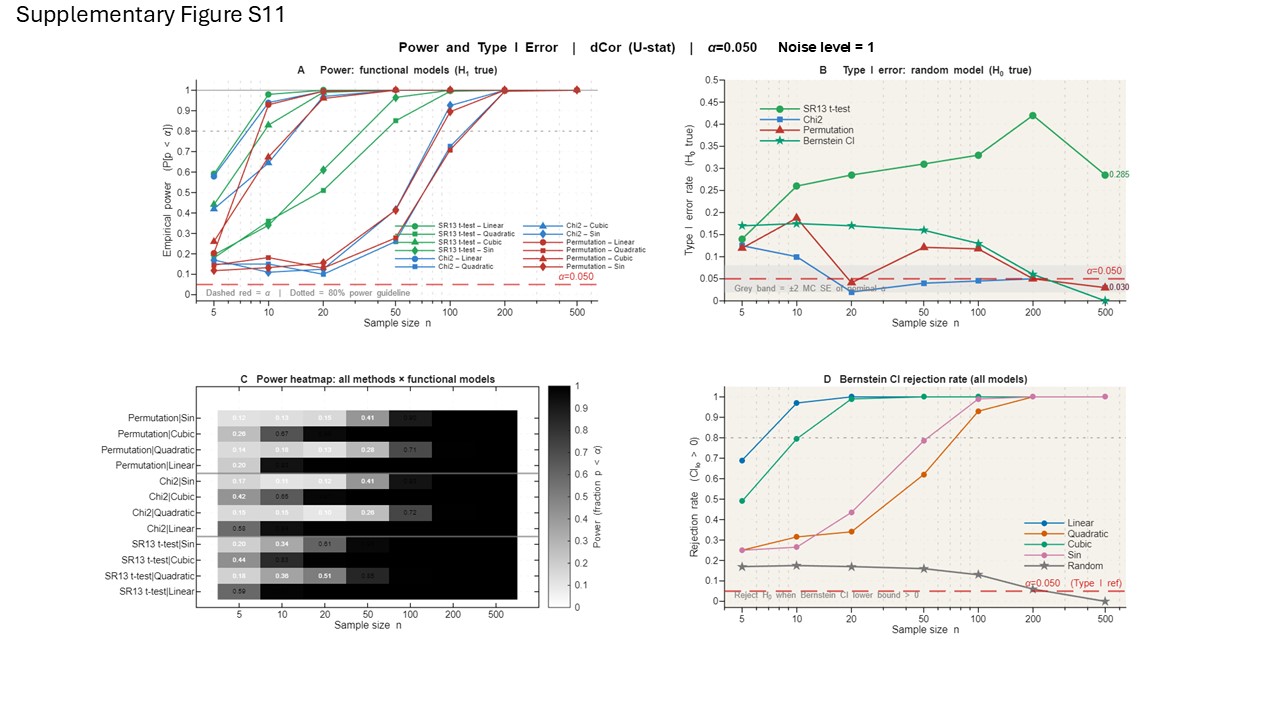

Supplement: Supplementary file 13 [file Image11.jpeg]

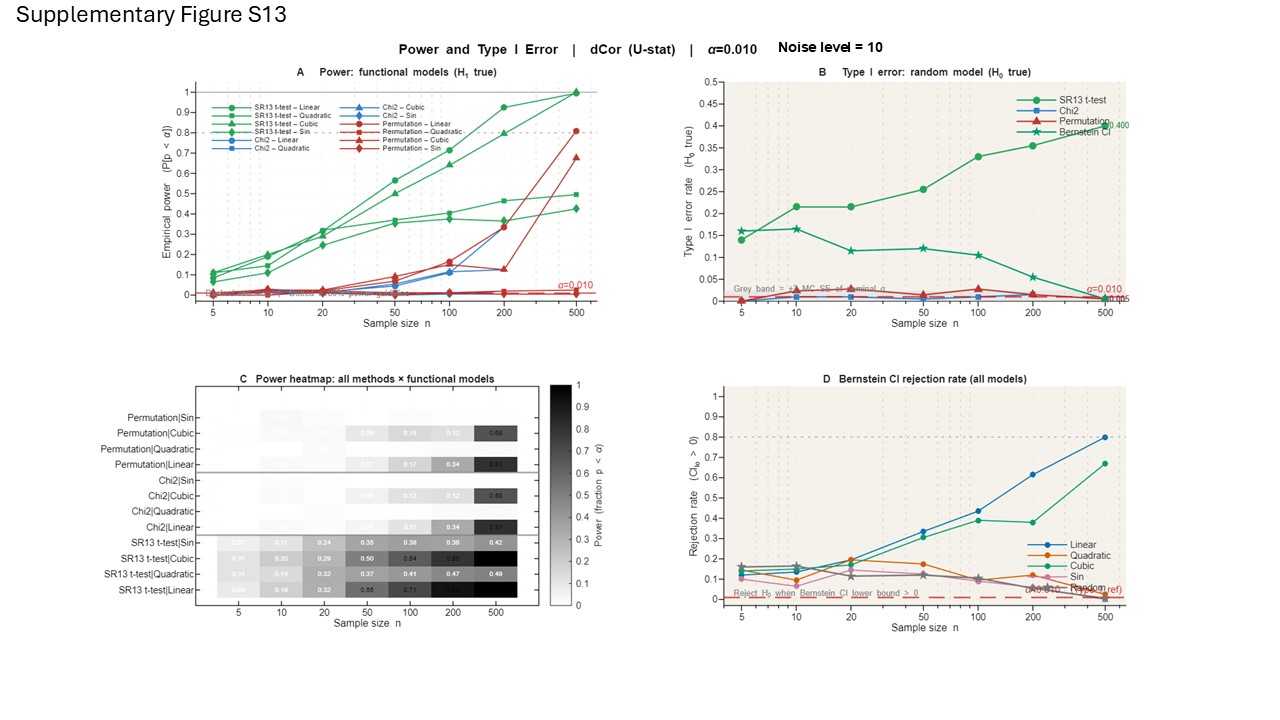

Supplement: Supplementary file 14 [file Image13.jpeg]

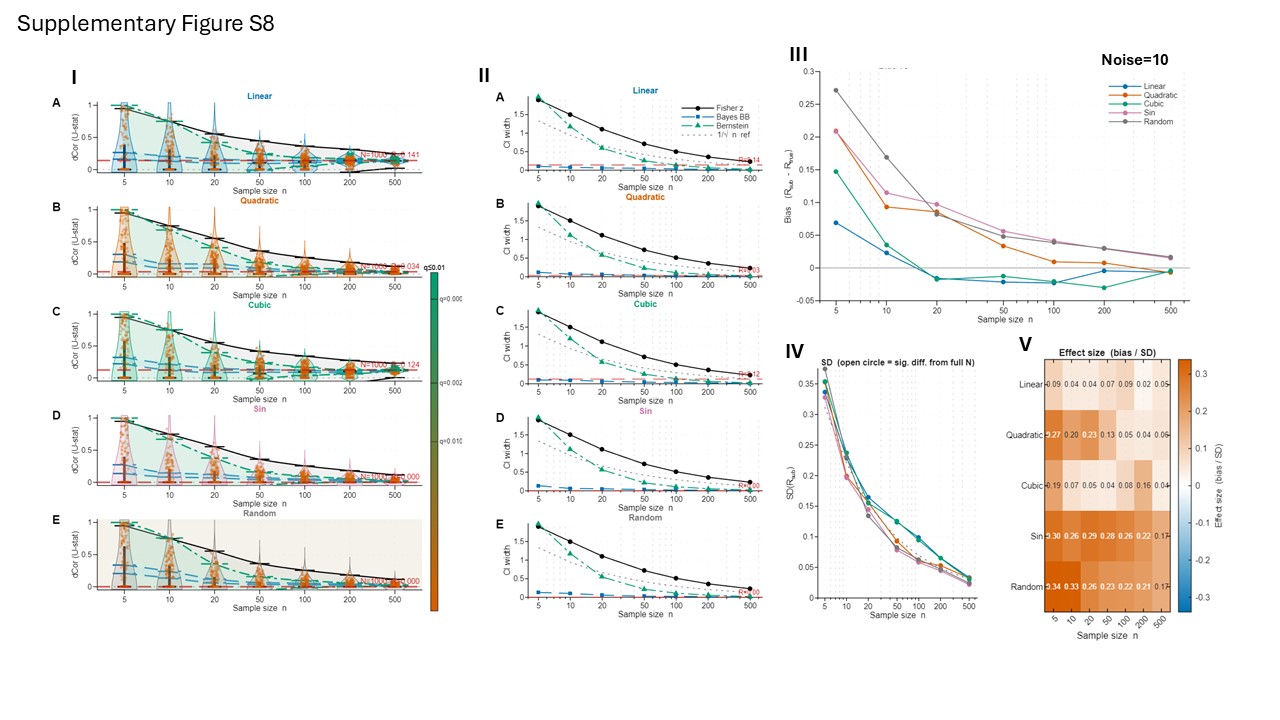

Supplement: Supplementary file 15 [file Image8.jpeg]

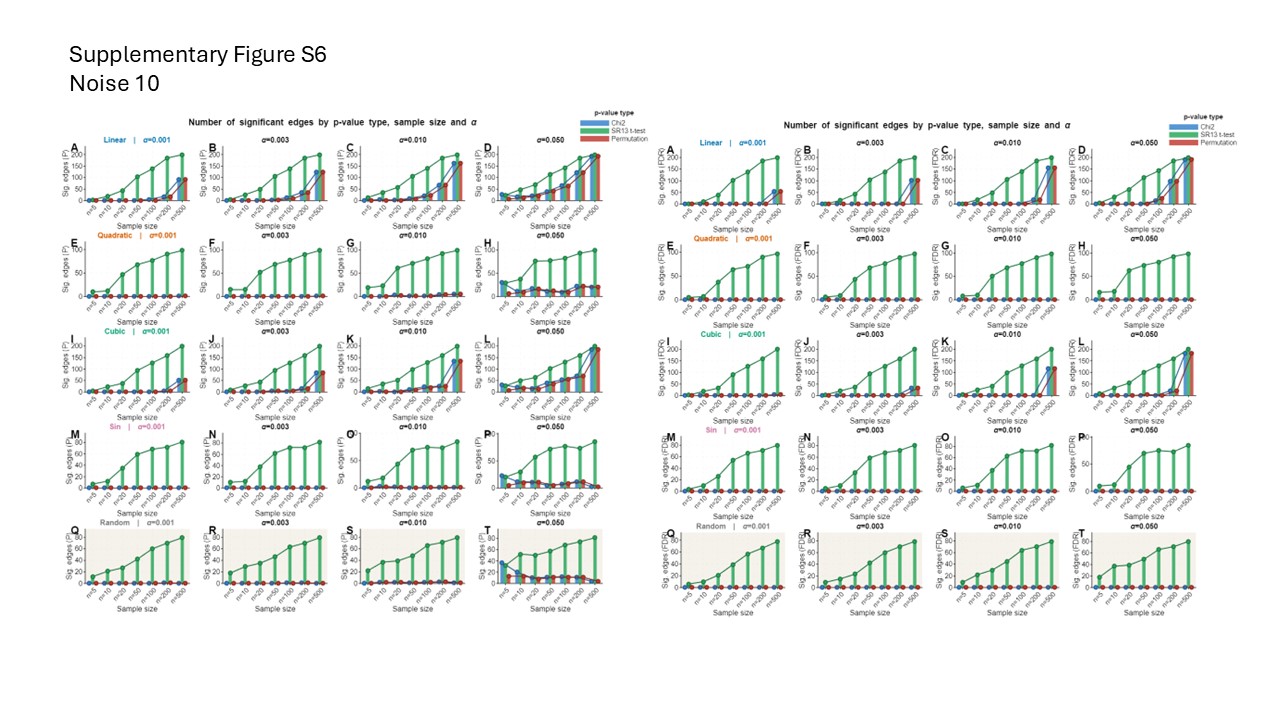

Supplement: Supplementary file 16 [file Image6.jpeg]
