# Supplementary material for: Unbiased distance correlation with sample-size-aware confidence bounds for comparative omics network analysis
Source: Front Bioinform. 2026 Jun 11;6:1788010. doi: 10.3389/fbinf.2026.1788010 (PMC13294210; doi:10.3389/fbinf.2026.1788010)
Supplement: Supplementary file 3 [file Supplementaryfile1.docx]

***Supplementary Materials***

**Supplementary Figures Index:**

**Supp. Figure S1 – Representation of simulation dataset 2**

**Supp. Figure S2 – Correlations and p-values sample dependence (Noise 0.1) 3**

**Supp. Figure S3 – Correlations and p-values sample dependence (Noise 10) 3**

**Supp. Figure S4 – Comparison of different p-value methods (Noise 0.1 and 10) 4**

**Supp. Figure S5 – Significant correlation pairs sample size dependence (Noise 0.1) 5**

**Supp. Figure S6 – Significant correlation pairs sample size dependence (Noise 10) 5**

**Supp. Figure S7 – Comparison of different CI approaches (Noise 0.1) 6**

**Supp. Figure S8 – Comparison of different CI approaches (Noise 10) 7**

**Supp. Figure S9 – Power and Type I error analysis (Noise 1, alpha=0.003) 8**

**Supp. Figure S10 – Power and Type I error analysis (Noise 1, alpha=0.01) 9**

**Supp. Figure S11 – Power and Type I error analysis (Noise 1, alpha=0.05) 10**

**Supp. Figure S12 – Power and Type I error analysis (Noise 0.1, alpha=0.01) 11**

**Supp. Figure S13 – Power and Type I error analysis (Noise 10, alpha=0.01) 12**

**Supp. Figure S14 – Scatter plot representation of network differences in SIDCO+ 13**

**Supp. Figure S15 – DCor network for Gln in NC and AD, SIDCO+ representation 13**


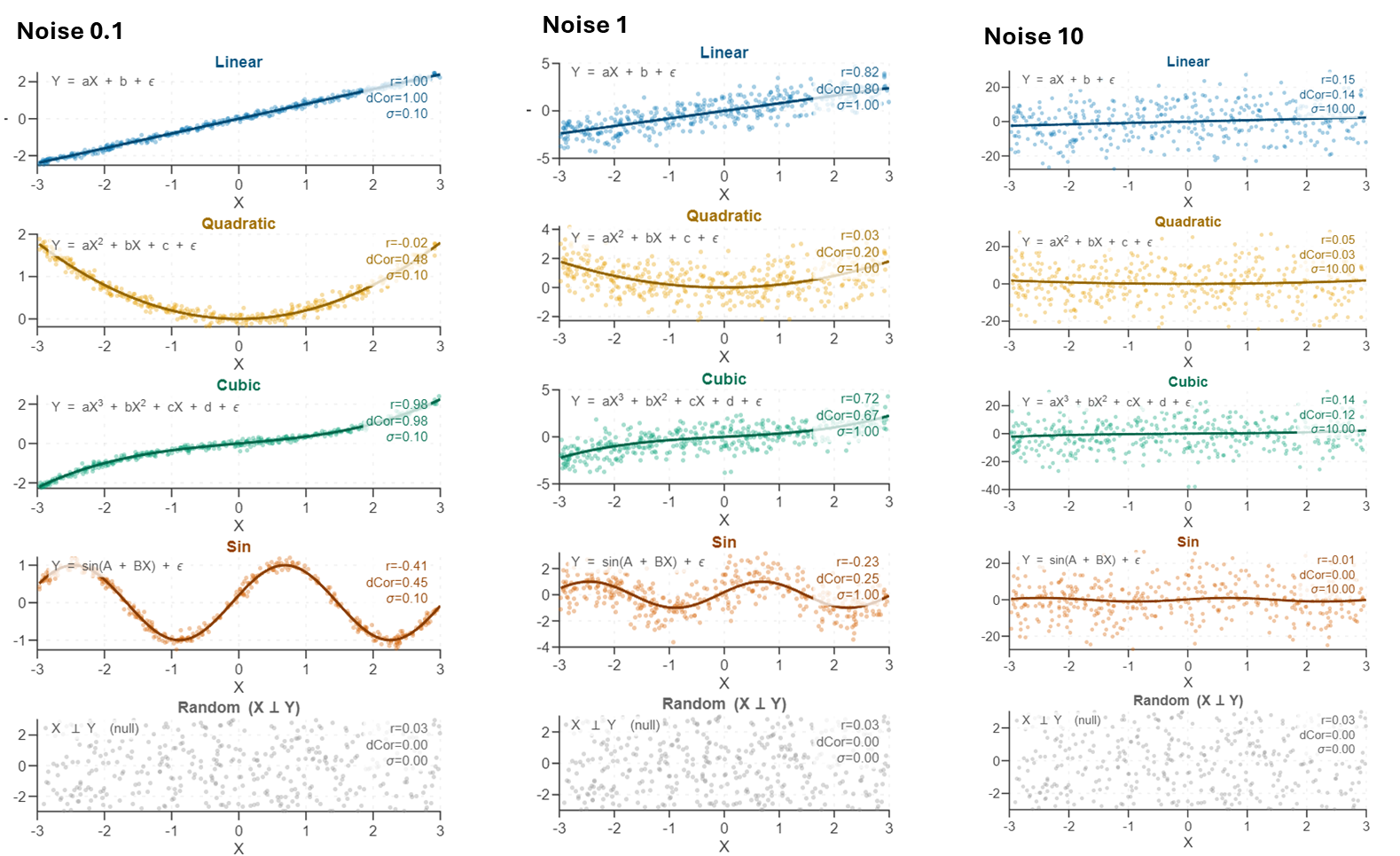
**Supplementary Figure S1**. Monte Carlo simulation datasets used in analysis. Three different noise levels were used to simulate high, medium and low correlation levels with noise multiplier set to 0.1, 1 and 10. In main publication all figures show intermediate noise level of 1. For each set simulated are 1000 samples. On each subfigure indicated is: equation used for simulating Y values from random values in X, r- Pearsons and dCor – biased distance correlation value for the simulated set and σ – added noise level for the given analysis.


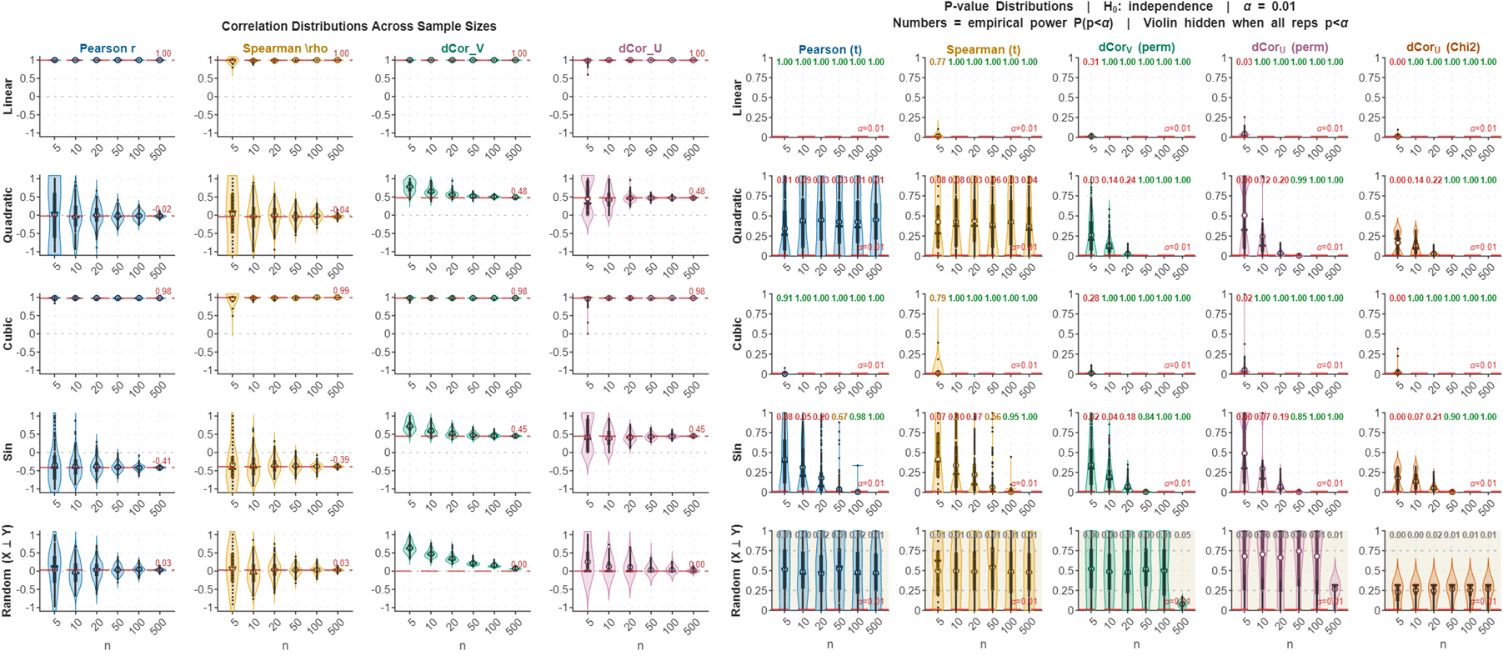
**Supplementary Figure S2.** 200 randomly selected subsets of samples of different size are selected from simulated 1000 points with correlation values calculated using 4 different methods. Examples show values at low noise (0.1) in the Monte Carlo simulations. Shown are correlation and p-values for Pearson, Spearman, biased distance correlation (dCor) and unbiased distance correlation (dCor_U). p-values are calculated using standard t-test method for Pearson and Spearman correlation, permutation test (with 500 permutations) for dCor and dCor_U and Chi2 test for dCor_U.

**
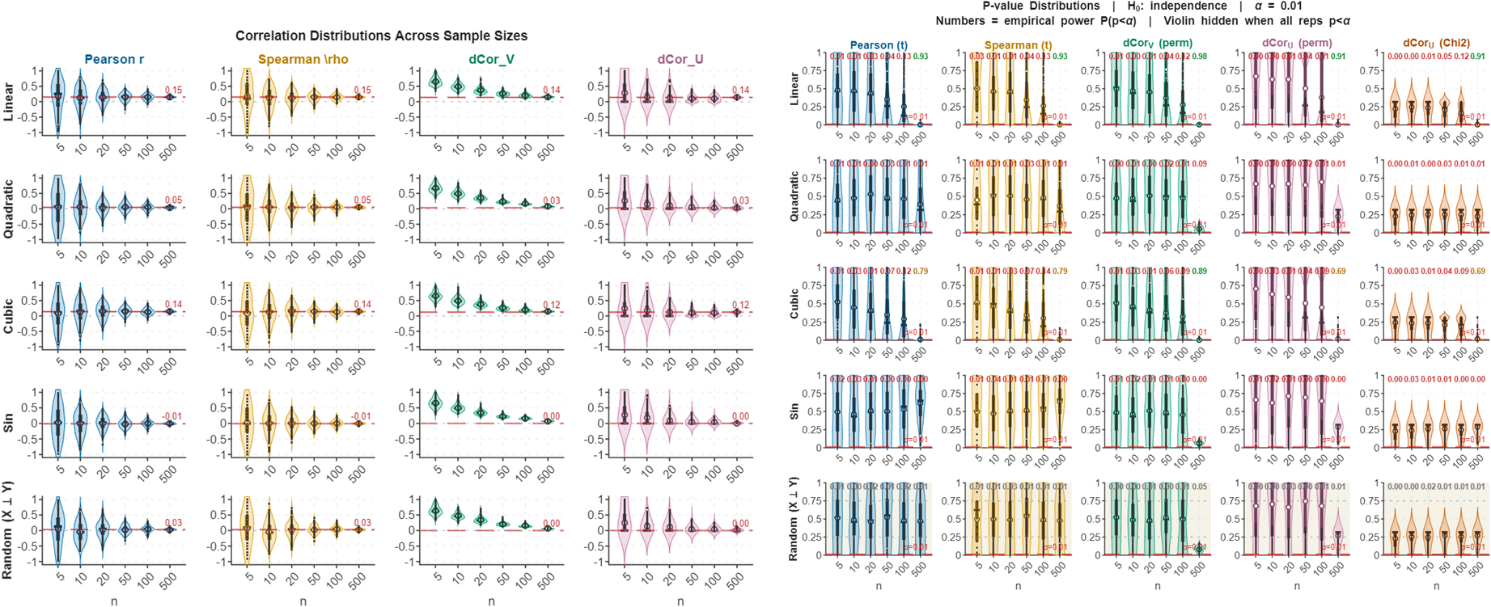
Supplementary Figure S3.** 200 randomly selected subsets of samples of different size are selected from simulated 1000 points with correlation values calculated using 4 different methods. Examples show values at high noise (10) in the Monte Carlo simulations. Shown are correlation and p-values for Pearson, Spearman, biased distance correlation (dCor) and unbiased distance correlation (dCor_U). p-values are calculated using standard t-test method for Pearson and Spearman correlation, permutation test (with 500 permutations) for dCor and dCor_U and Chi2 test for dCor_U.

**
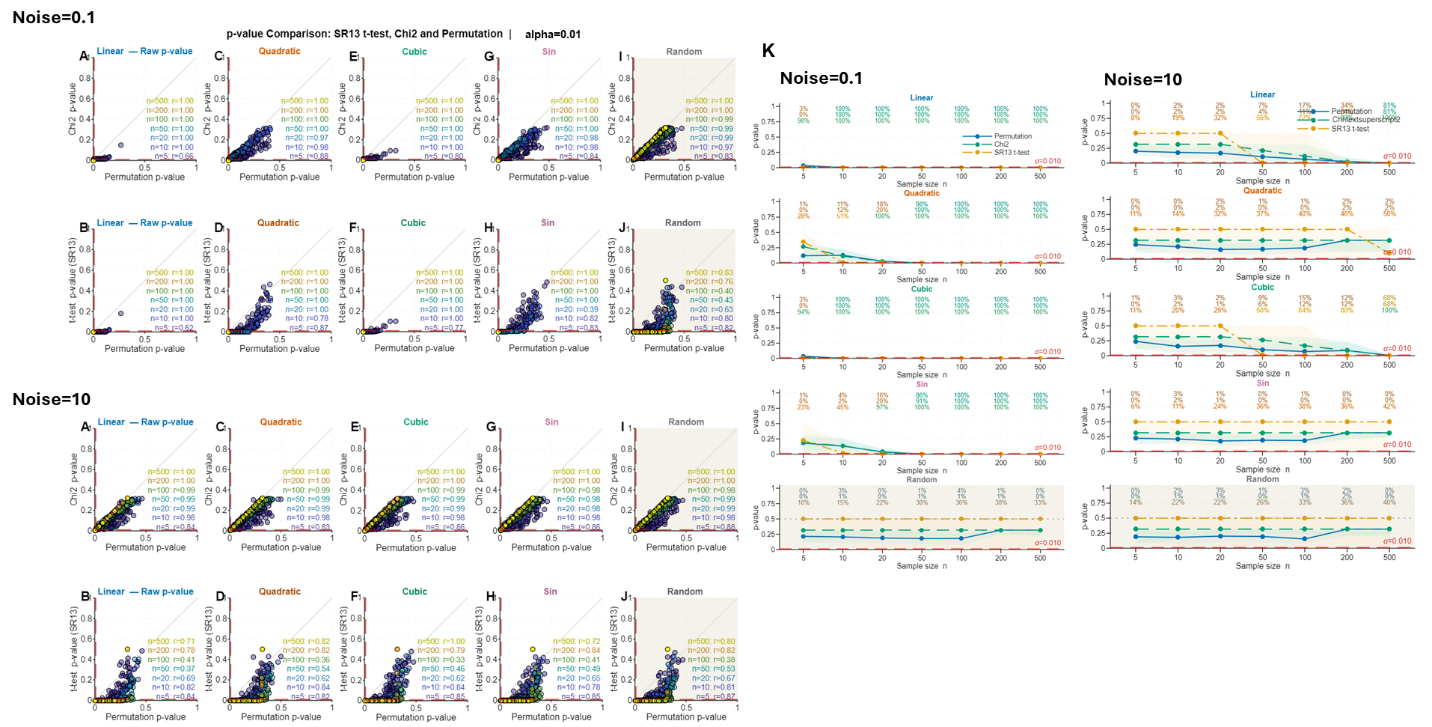
 Supplementary Figure S4.** Added is noise (multiplier 0.1 or 10) leading to intermediate strong correlations (plots for multiplier levels 0.1 and 10 are shown in Supplementary Figures). In all plots x-axis corresponds to: p-values obtained using permutation test with 500 permutations. For A-I y-axis shows values obtained using Chi2 method (Eq(11), following Shen et al. (2021)); In B-J plots y-axis shows p-values obtained using formula shown in Eq.(12) for the limit of high dimensionality presented by (Szekely, 2013). Gao et al. (2021). Included is information about the number of points with both p-values below the alpha=0.01 threshold (r value for each sample size). r =1 indicates that methods fully agree about points that are statistically significant. K. Change in p-value for three methods with sample size at different functional dependencies.

**
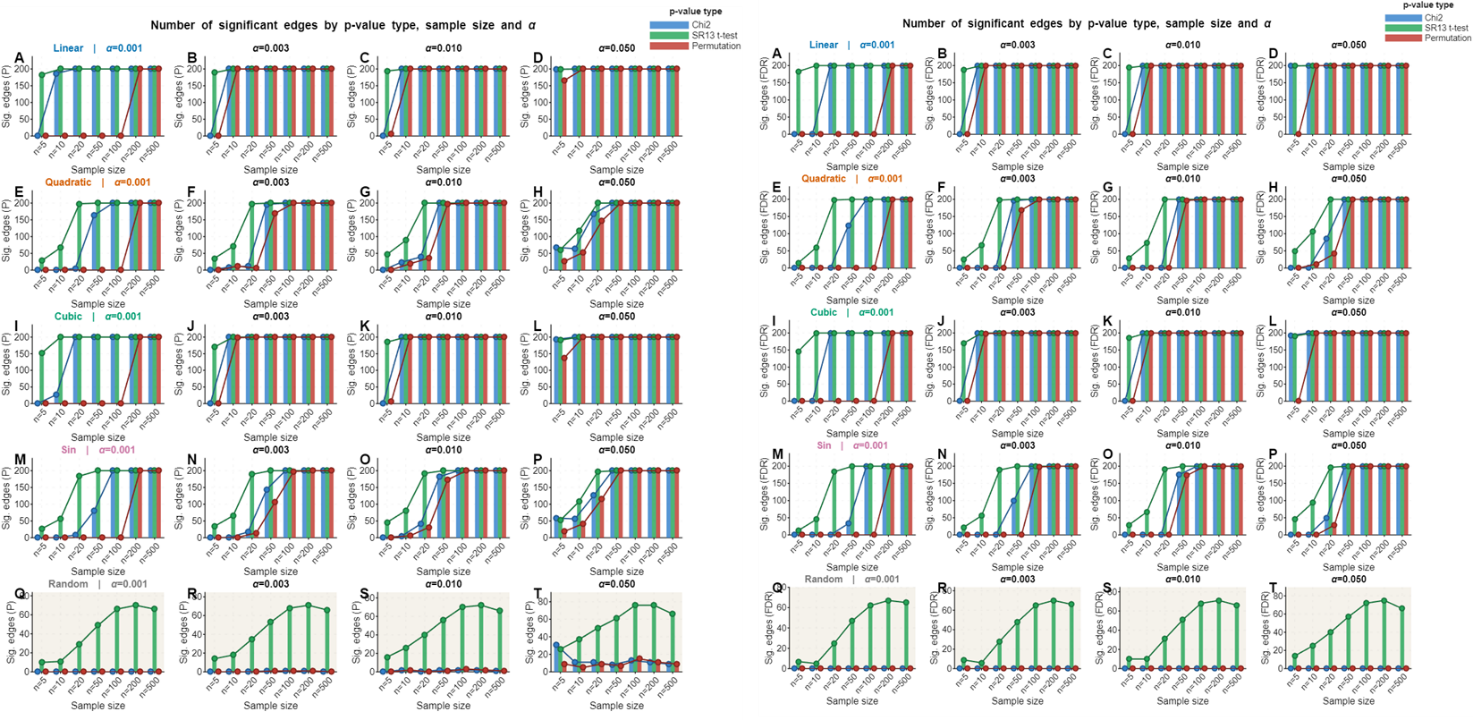
Supplementary Figure S5.** Number of correlation pairs that pass different significant level threshold based on three different p-value calculation methods without (I) or with FDR (II) correction for the sets simulated with noise level 0.1.

**
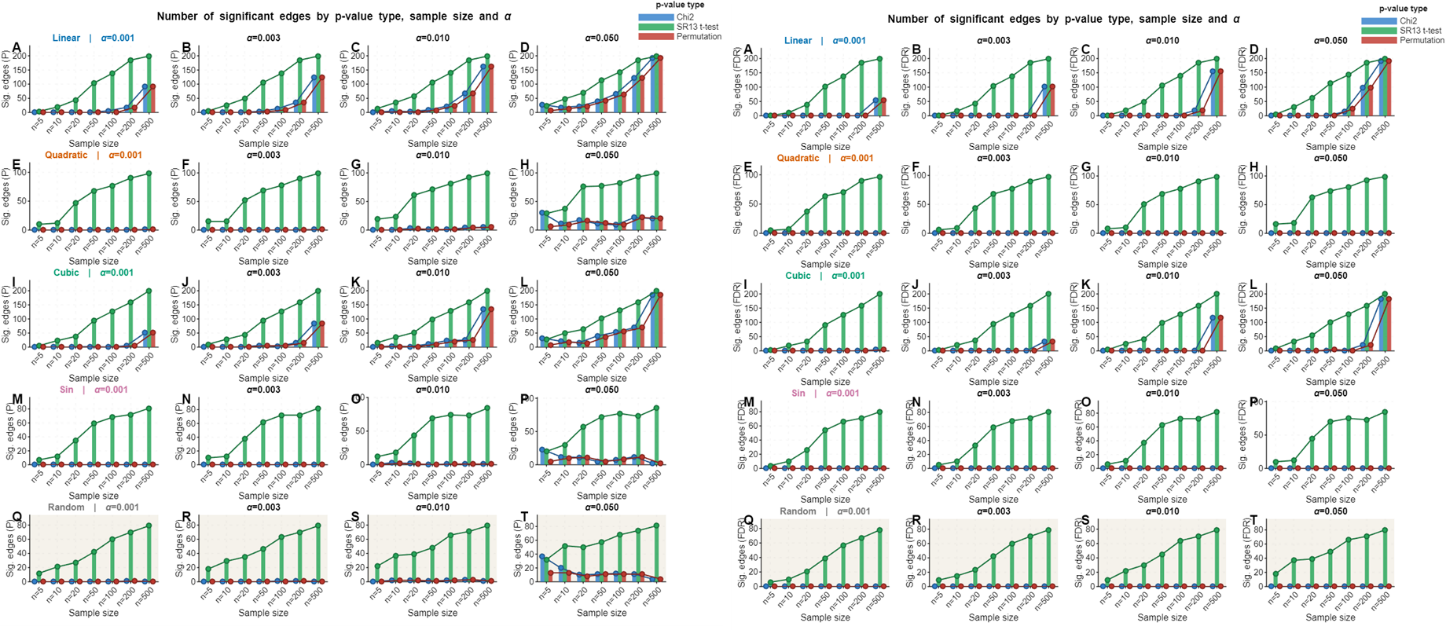
Supplementary Figure S6.** Number of correlation pairs that pass different significant level points based on three different p-value calculation methods without (I) or with FDR (II) for the sets simulated with noise level 10.

**
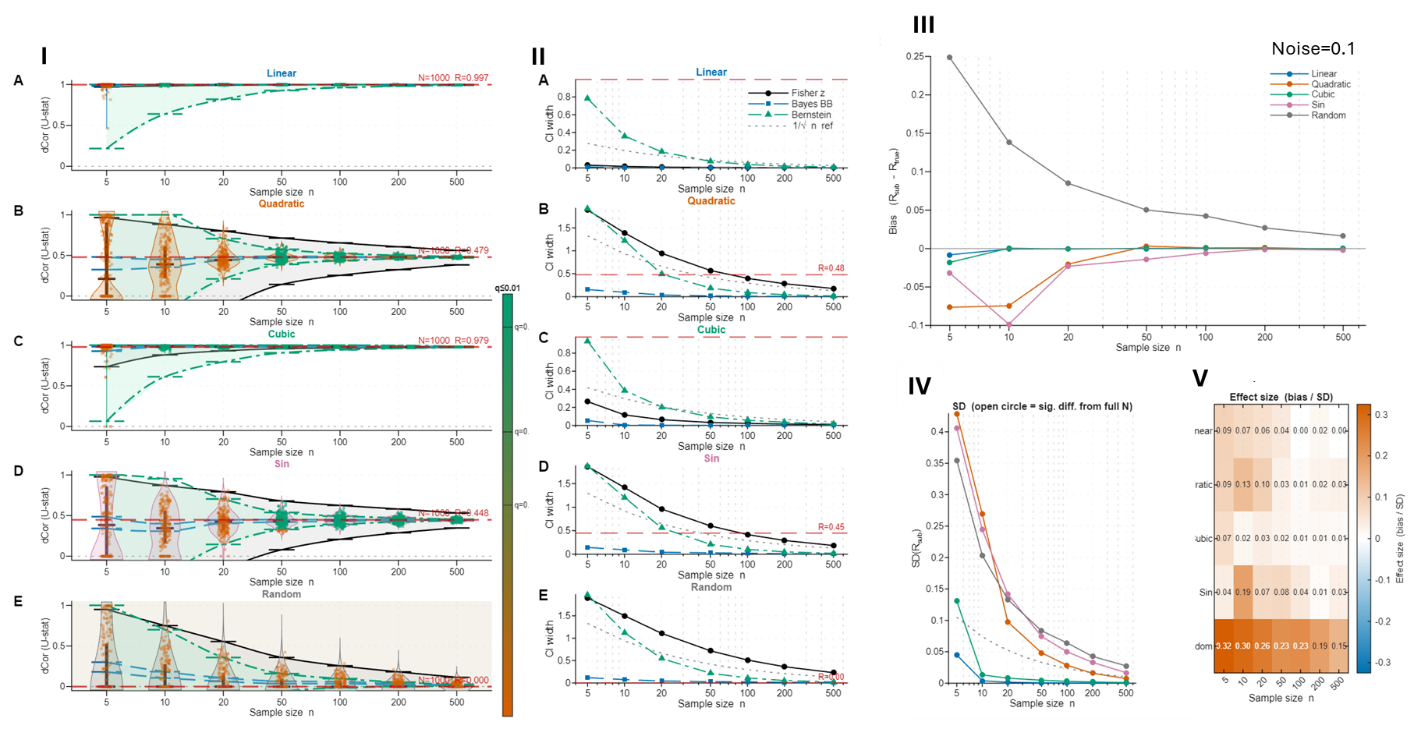
Supplementary Figure S7.** shows Monte Carlo simulation of Distance correlation values with CI using three different methods for Monte Carlo simulated dataset with noise multiplier=0.1 I. Panels show values for 200 runs with replacement at different sample sizes. In green are values that pass statistical significance threshold (0.01) with lines for CI boundaries indicated for Fisher (black), Bayes Bootstrap (blue) and Bernstein (green) indicated; II. Upper CI boundaries of the three methods. III. Bias of correlation values for sample size *n* compared to sample size *N*=1000, complete set. Shown is bias for different functional dependencies including random values; IV. Standard deviation of obtained dCor values at different sample sizes and for tested functional dependences; V. Effect size comparing values for different sample sizes against *N*=1000 calculated as Cohen’s d-average ratio of bias and mean SD (Lakens, 2013).

**
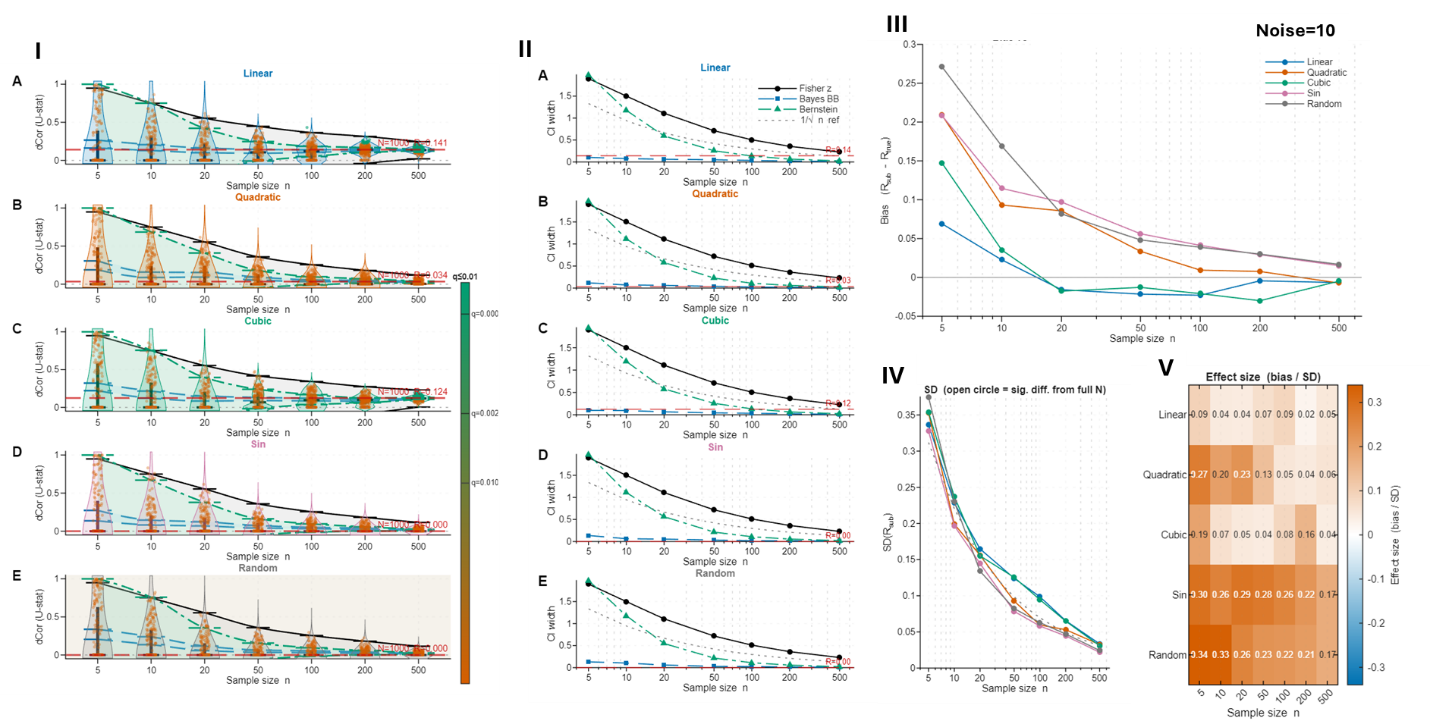
Supplementary Figure S8.** shows Monte Carlo simulation of Distance correlation values with CI using three different methods for Monte Carlo simulated dataset with noise multiplier=10; I. Panels show values for 200 runs with replacement at different sample sizes. In green are values that pass statistical significance threshold (0.01) with lines for CI boundaries indicated for Fisher (black), Bayes Bootstrap (blue) and Bernstein (green) indicated; II. Upper CI boundaries of the three methods. III. Bias of correlation values for sample size *n* compared to sample size *N*=1000, complete set. Shown is bias for different functional dependencies including random values; IV. Standard deviation of obtained dCor values at different sample sizes and for tested functional dependences; V. Effect size comparing values for different sample sizes against *N*=1000 calculated as Cohen’s d-average ratio of bias and mean SD (Lakens, 2013).


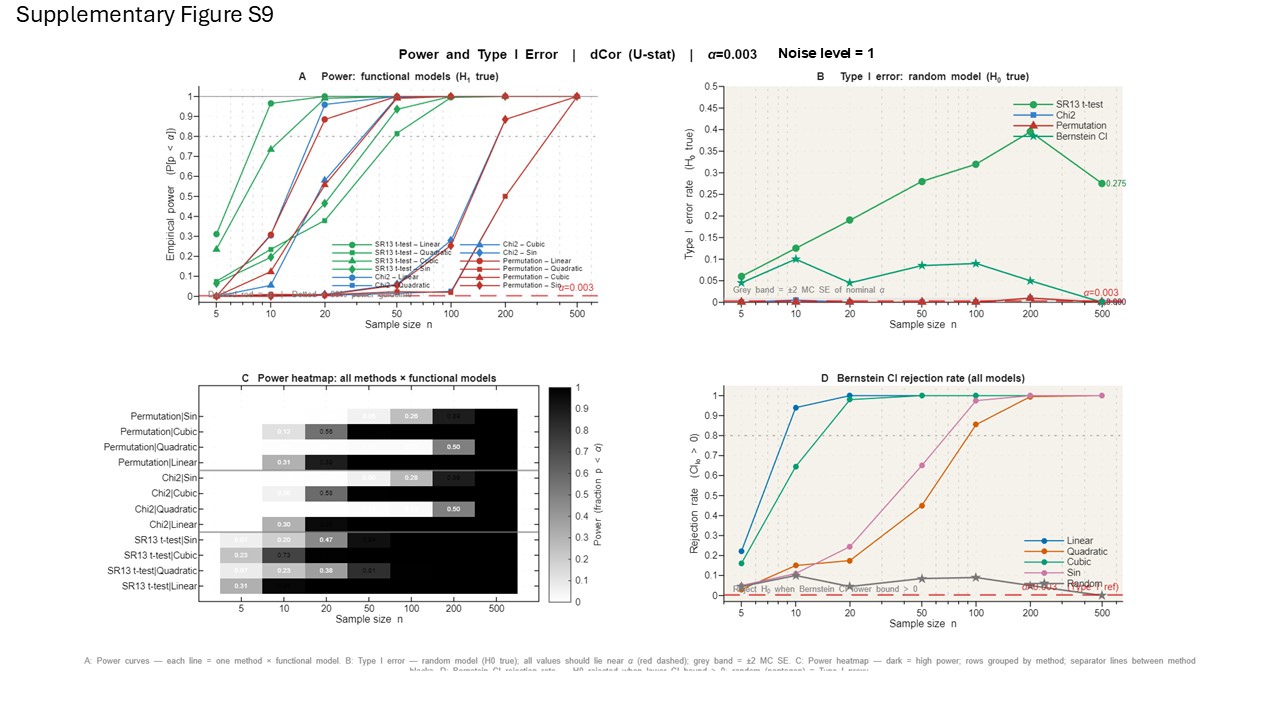


**Supplementary Figure S9.** Further representation of power and Type I error analysis for the simulation models. Shown are dependencies at Noise level 1 and alpha=0.003. Panels are: A. Empirical power analysis showing the proportion of trials in a simulation for each functional model where a statistical test rejects a false null hypothesis, calculated as the frequency of statistically significant results for three different p-value calculation methods – permutation, Chi-square and t-test based method. B. Type I error rate as a proportion of not rejected tests in random set (no correlation case). Shown are again proportions from three different p-value calculation methods and Bernstein CI test at low CI value of 0.1; C. Power heatmap providing direct comparison between different m p-value calculation methods and functional sets in terms of empirical power; D. Empirical power analysis for Bernstein CI with limit for low CI >0.1.


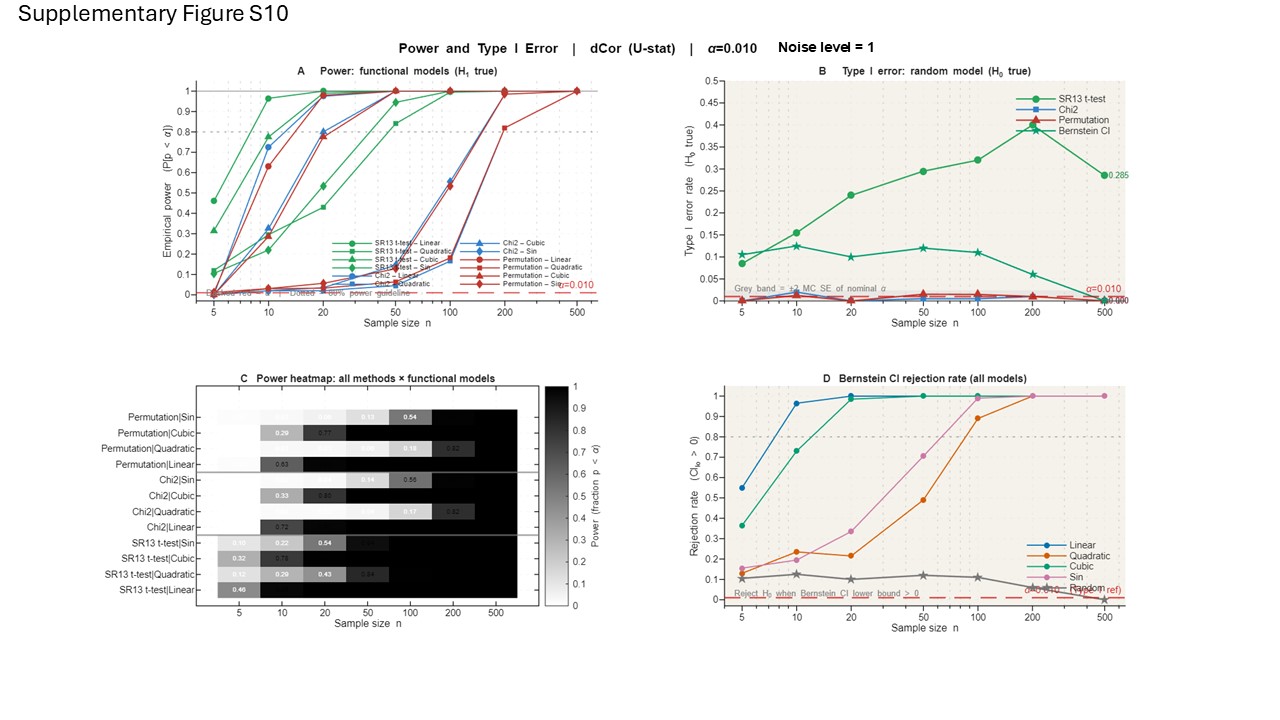


**Supplementary Figure S10.** Further representation of power and Type I error analysis for the simulation models. Shown are dependencies at Noise level 1 and alpha=0.01. Panels are: A. Empirical power analysis showing the proportion of trials in a simulation for each functional model where a statistical test rejects a false null hypothesis, calculated as the frequency of statistically significant results for three different p-value calculation methods – permutation, Chi-square and t-test based method. B. Type I error rate as a proportion of not rejected tests in random set (no correlation case). Shown are again proportions from three different p-value calculation methods and Bernstein CI test at low CI value of 0.1; C. Power heatmap providing direct comparison between different m p-value calculation methods and functional sets in terms of empirical power; D. Empirical power analysis for Bernstein CI with limit for low CI >0.1.


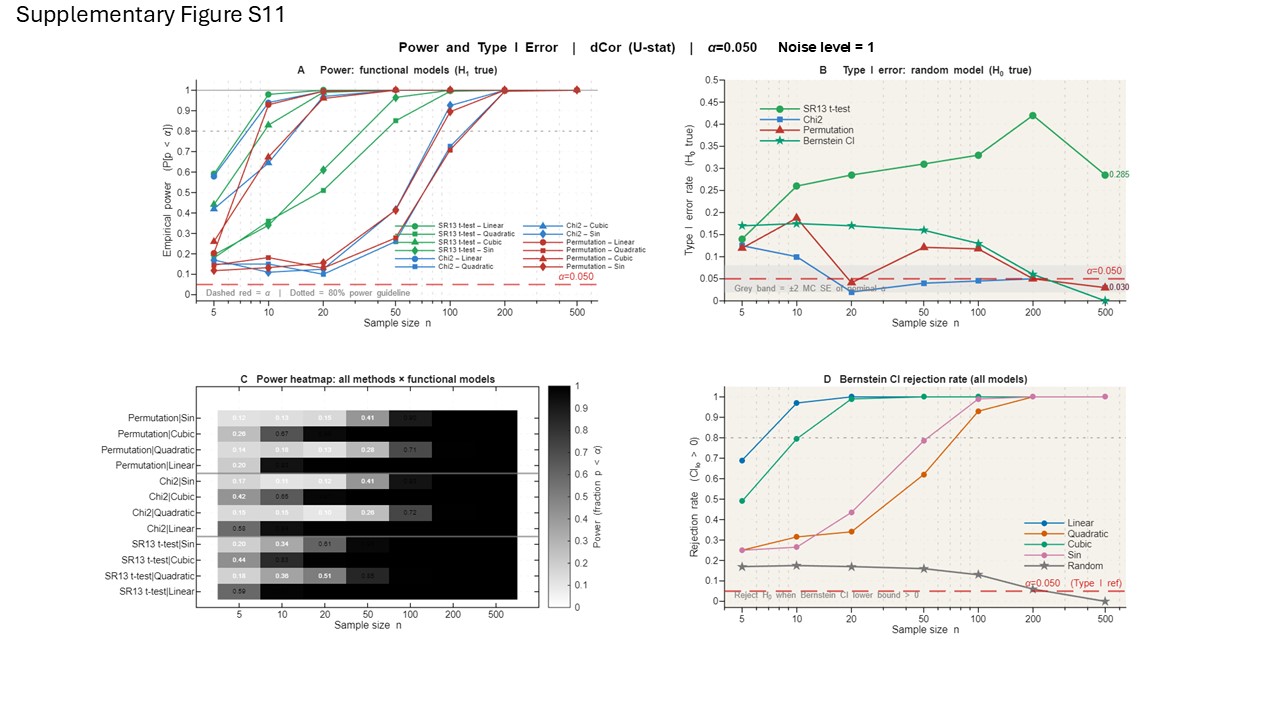
 **Supplementary Figure S11.** Further representation of power and Type I error analysis for the simulation models. Shown are dependencies at Noise level 1 and alpha=0.05. Panels are: A. Empirical power analysis showing the proportion of trials in a simulation for each functional model where a statistical test rejects a false null hypothesis, calculated as the frequency of statistically significant results for three different p-value calculation methods – permutation, Chi-square and t-test based method. B. Type I error rate as a proportion of not rejected tests in random set (no correlation case). Shown are again proportions from three different p-value calculation methods and Bernstein CI test at low CI value of 0.1; C. Power heatmap providing direct comparison between different m p-value calculation methods and functional sets in terms of empirical power; D. Empirical power analysis for Bernstein CI with limit for low CI >0.1.


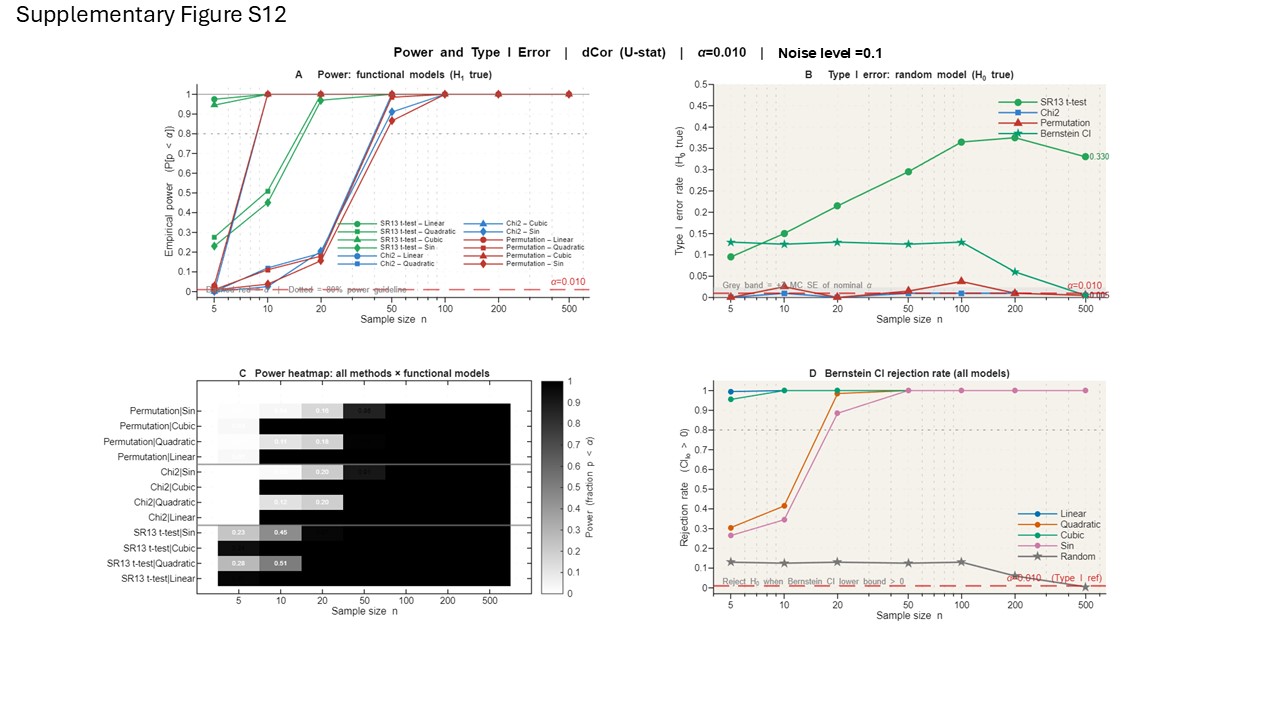


**Supplementary Figure S12.** Further representation of power and Type I error analysis for the simulation models. Shown are dependencies at Noise level 0.1 and alpha=0.01. Panels are: A. Empirical power analysis showing the proportion of trials in a simulation for each functional model where a statistical test rejects a false null hypothesis, calculated as the frequency of statistically significant results for three different p-value calculation methods – permutation, Chi-square and t-test based method. B. Type I error rate as a proportion of not rejected tests in random set (no correlation case). Shown are again proportions from three different p-value calculation methods and Bernstein CI test at low CI value of 0.1; C. Power heatmap providing direct comparison between different m p-value calculation methods and functional sets in terms of empirical power; D. Empirical power analysis for Bernstein CI with limit for low CI >0.1.


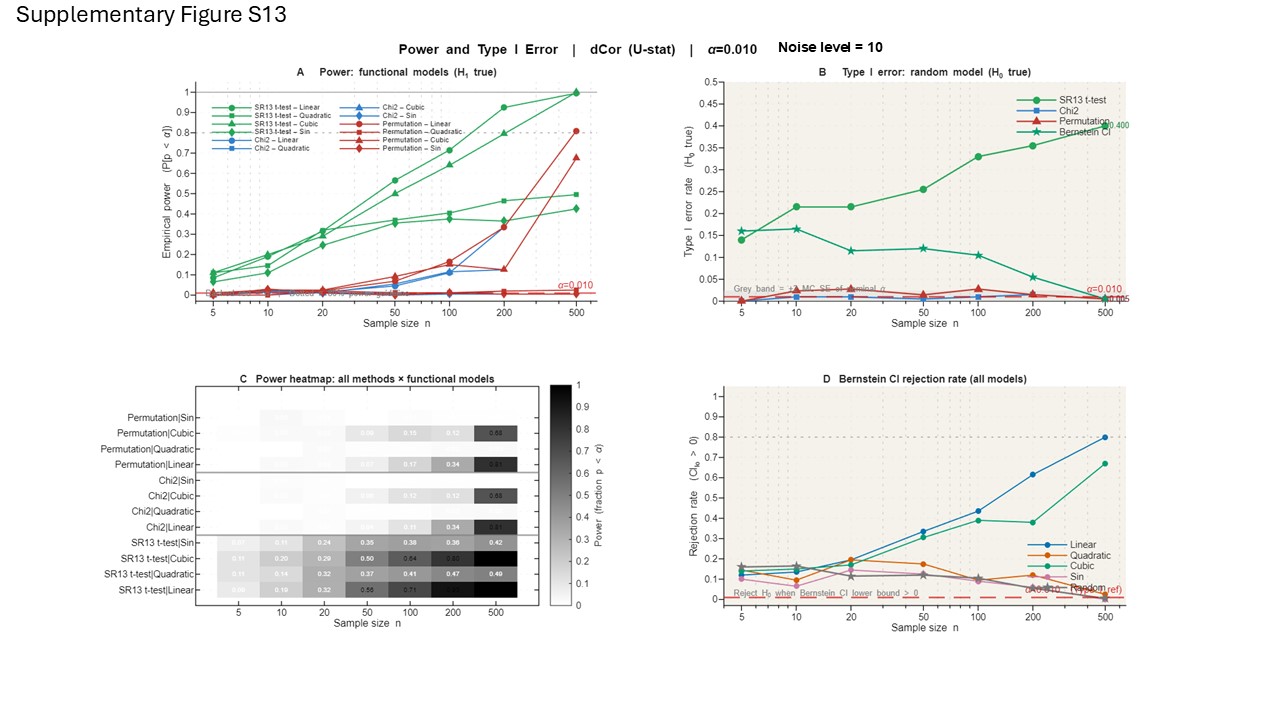
 **Supplementary Figure S13.** Further representation of power and Type I error analysis for the simulation models. Shown are dependencies at Noise level 10 and alpha=0.01. Panels are: A. Empirical power analysis showing the proportion of trials in a simulation for each functional model where a statistical test rejects a false null hypothesis, calculated as the frequency of statistically significant results for three different p-value calculation methods – permutation, Chi-square and t-test based method. B. Type I error rate as a proportion of not rejected tests in random set (no correlation case). Shown are again proportions from three different p-value calculation methods and Bernstein CI test at low CI value of 0.1; C. Power heatmap providing direct comparison between different m p-value calculation methods and functional sets in terms of empirical power; D. Empirical power analysis for Bernstein CI with limit for low CI >0.1.


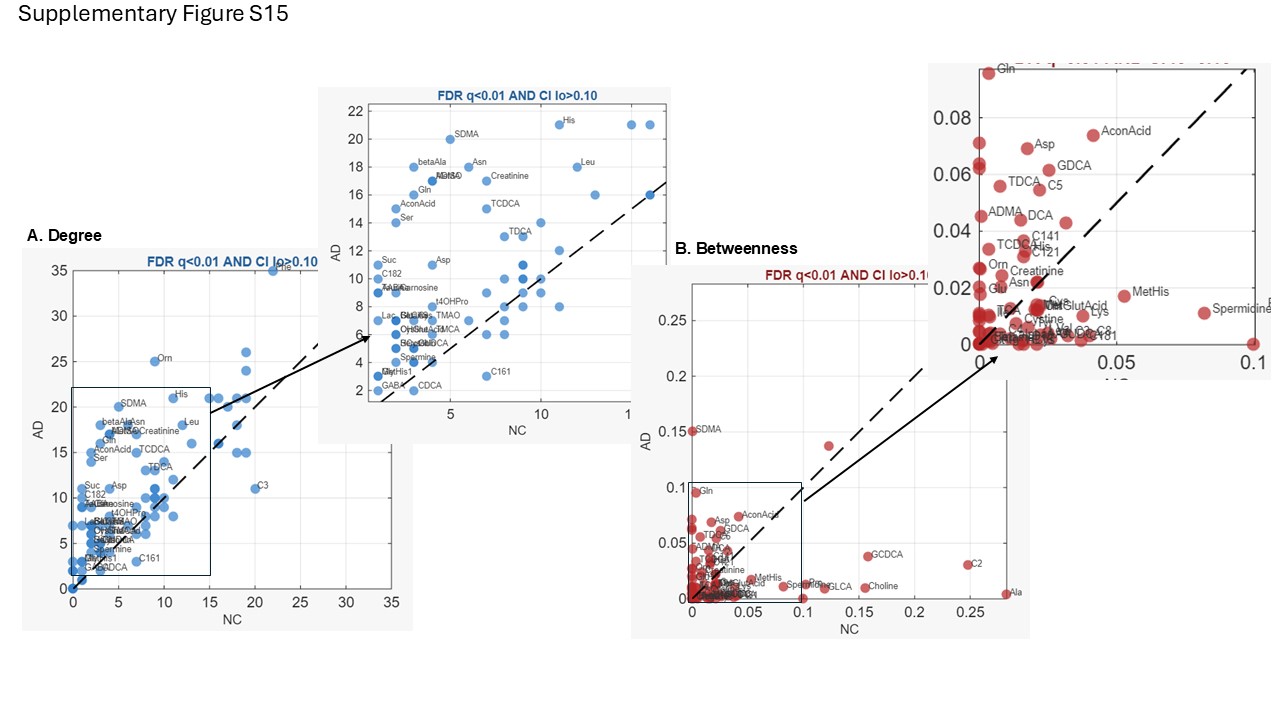


**Supplementary Figure S14.** Scatter plot representation of the difference between degree and betweenness for nodes in NC and AD datasets. In both cases metabolite label is shown when difference is more than 50% between values in two sets.


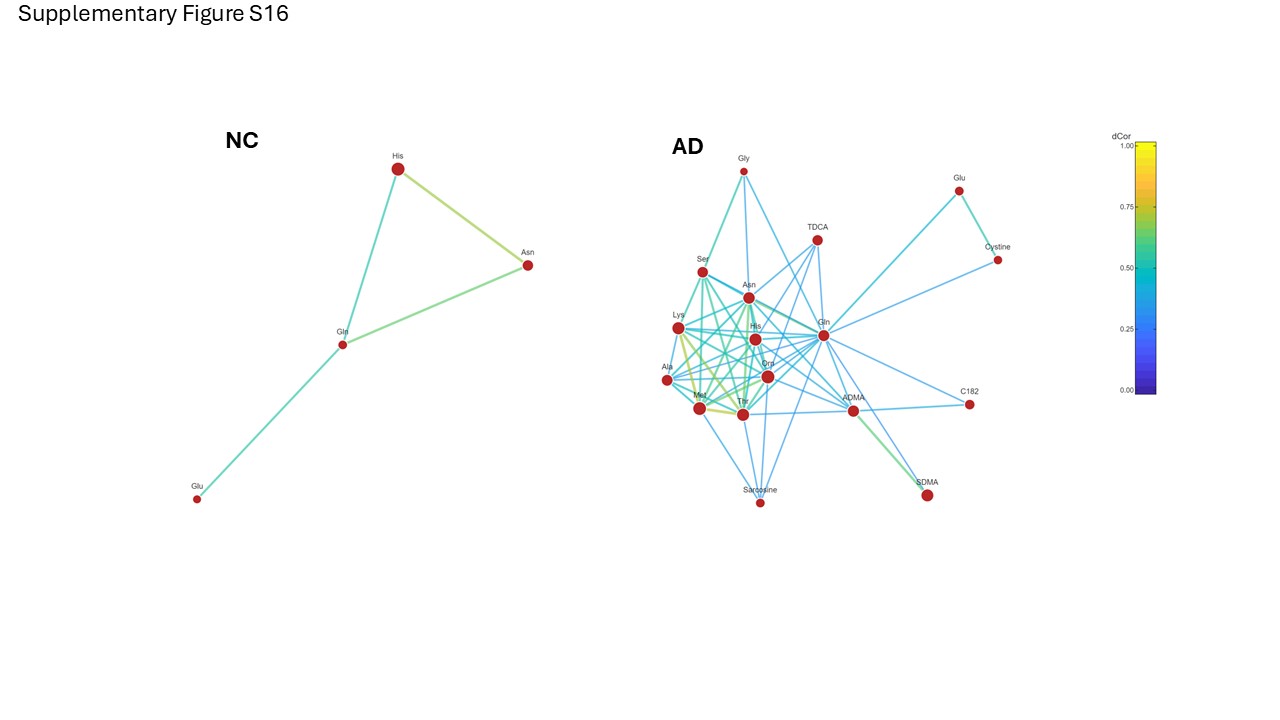


**Supplementary Figure S15.** Significant correlation network for glutamine (Gln) in Control samples (NC) and Alzheimer disease group (AD).
